# Miz-1 Activates Gene Expression via a Novel Consensus DNA Binding Motif

**DOI:** 10.1371/journal.pone.0101151

**Published:** 2014-07-01

**Authors:** Bonnie L. Barrilleaux, Dana Burow, Sarah H. Lockwood, Abigail Yu, David J. Segal, Paul S. Knoepfler

**Affiliations:** 1 Department of Cell Biology and Human Anatomy, University of California Davis School of Medicine, Davis, California, United States of America; 2 Genome Center, University of California Davis, Davis, California, United States of America; 3 Comprehensive Cancer Center, University of California Davis, Sacramento, California, United States of America; 4 Department of Biochemistry, University of California Davis, Davis, California, United States of America; 5 Institute of Pediatric Regenerative Medicine, Shriners Hospital For Children Northern California, Sacramento, California, United States of America; University of Manchester, United Kingdom

## Abstract

The transcription factor Miz-1 can either activate or repress gene expression in concert with binding partners including the Myc oncoprotein. The genomic binding of Miz-1 includes both core promoters and more distal sites, but the preferred DNA binding motif of Miz-1 has been unclear. We used a high-throughput *in vitro* technique, Bind-n-Seq, to identify two Miz-1 consensus DNA binding motif sequences—ATCGGTAATC and ATCGAT (Mizm1 and Mizm2)—bound by full-length Miz-1 and its zinc finger domain, respectively. We validated these sequences directly as high affinity Miz-1 binding motifs. Competition assays using mutant probes indicated that the binding affinity of Miz-1 for Mizm1 and Mizm2 is highly sequence-specific. Miz-1 strongly activates gene expression through the motifs in a Myc-independent manner. MEME-ChIP analysis of Miz-1 ChIP-seq data in two different cell types reveals a long motif with a central core sequence highly similar to the Mizm1 motif identified by Bind-n-Seq, validating the *in vivo* relevance of the findings. Miz-1 ChIP-seq peaks containing the long motif are predominantly located outside of proximal promoter regions, in contrast to peaks without the motif, which are highly concentrated within 1.5 kb of the nearest transcription start site. Overall, our results indicate that Miz-1 may be directed *in vivo* to the novel motif sequences we have identified, where it can recruit its specific binding partners to control gene expression and ultimately regulate cell fate.

## Introduction

Miz-1 (*ZBTB17*) is a BTB/POZ (BR-C, ttk and bab/pox virus and zinc-finger) domain-containing transcription factor that is ubiquitously expressed throughout development. It was named via an acronym for “Myc-interacting zinc finger” protein. Miz-1 was originally functionally characterized as an inducer of growth arrest [1]. Subsequently, Miz-1 was found to be critical in normal development [2]–[4] and to play roles in human disease [5], [6]. Miz-1 binds core promoters to activate target genes [7]. However, in the presence of the basic helix-loop-helix transcription factor, Myc, the function of Miz-1 shifts from activation to repression of transcription. Myc and Miz-1 form a co-repressor complex, silencing Miz-1 target genes including those associated with differentiation and proliferation [8]. Thus, there exist both Myc-dependent and Myc-independent functions of Miz-1. Still, relatively little is known about the function of Miz-1 as a transcriptional regulator.

Miz-1 can form a co-repressor complex with Myc by binding through its Myc interaction domain, amino acids 683–715, to silence Miz-1 target genes [1]. Myc-Miz interaction represses Miz-1 gene activation at least in part by competing with the co-activator p300 [7]. Recent genomics studies in stem cells also support the hypothesis that the mechanism by which Myc normally represses expression of differentiation genes, thereby maintaining pluripotency and self-renewal, is at least in part mediated via coordinated function with Miz-1 [9]. Alternately, Miz-1 can form a co-activating complex with p300 and NPM, activating target genes in a Myc-independent fashion [10], [11]. Genome-wide chromatin immunoprecipitation-microarray (ChIP-chip) analysis indicates that Myc occupies nearly 30% of Miz-1 targets in human embryonic stem cells (hESCs), while about 70% of Miz-1 targets are not co-bound by Myc [9]. Interestingly and contrary to previous studies that analyzed Miz-1 regulation of specific candidate genes [7], [8], [10], [12]–[14], which described Miz-1 binding localized to core promoter initiator element (Inr) sequences in cancer cells, the global functional genomics analysis in hESCs demonstrated that the distribution of Miz-1 binding is predominantly localized to regions more than 1000 bases upstream of the transcriptional start sites of target genes [9]. Conversely, a recent study reports Miz-1 binding predominantly at proximal promoters in murine neural progenitor cells [15]. The authors report a consensus binding motif in mouse cells, but so far no specific DNA binding motif for Miz-1 in human cells has been identified. Thus, identification of human Miz-1 consensus DNA binding motifs is central to understanding the genomic binding of Miz-1 and its regulation of cellular biology.

Using a maltose binding protein (MBP) fusion protein tag system, we employed Bind-n-Seq (BnS), an *in vitro*, high-throughput DNA binding assay with Multiple EM for Motif Elicitation (MEME) analysis to identify putative Miz-1 DNA biding motifs *de novo*. The BnS method is an efficient and comprehensive way to examine protein-DNA binding in a global, unbiased manner [16]. BnS overcomes problems associated with other motif-finding approaches including limitations on *in vivo* detection and sensitivity, and time and labor-intensive *in vitro* approaches. Instead, BnS employs massively parallel sequencing of annealed oligonucleotides bound to MBP-tagged proteins.

We identified two novel putative Miz-1 consensus DNA binding motifs, ATCGGTAATC (Mizm1) and ATCGAT (Mizm2), through this BnS analysis. These motifs were then confirmed as Miz-1-bound using electrophoretic mobility shift assays (EMSA). Luciferase reporter assays demonstrated that Miz-1 can activate gene expression via the motifs. These motifs are biologically significant, bearing a strong resemblance to motifs that we identified in recently published mouse and human Miz-1 ChIP-seq data by another group. Interestingly, Mizm1 and Mizm2 are also similar to motifs bound by Cut homeodomain proteins including Cux1. However, we found that Cux1 and Miz-1 differ substantially in their relative preference for each motif, indicating that the similarity between the motifs is likely not functionally relevant. In this work we have identified a preferred DNA motif bound by Miz-1, and demonstrated its function in regulating transcription, indicating a potential mechanism for the Inr-independent genomic binding of Miz-1. Understanding the direct genomic binding of Miz-1 will help to further our knowledge of the transcriptional processes it directs and the effects on cell biology.

## Materials and Methods

### Recombinant Protein Expression and Purification

We generated a plasmid vector coding for an N-terminal fusion of *E. coli* maltose binding protein (MBP) to full-length human Miz-1 (MBP-Miz-1-FL) by restriction ligation of Miz-1 cDNA generated from H9 hESC mRNA into pMAL-c5G (New England Biolabs). To generate a fusion of MBP with the zinc finger domain of Miz-1 (MBP-Miz-1-ZF), we cloned the sequence encoding the 13 C2H2 zinc fingers (nucleotides 805–2379) of human Miz-1 in frame into a plasmid vector coding for an N-terminal MBP tag. Isopropyl β-D-1-thiogalactopyranoside (IPTG) was used to induce expression of MBP, MBP-Miz-1-FL, and MBP-Miz-1-ZF in E. coli (BL21STAR). Five hours after IPTG induction, cells were harvested by centrifugation (3500 rpm, 20 min, 4°C) and sonicated in Zinc Buffer A [ZBA; 10 mM Tris (pH 7.5), 90 mM KCl, 1 mM MgCl_2_, 90 µM ZnCl_2_, 5 mM DTT]. The resulting protein lysate was cleared by centrifugation (20,000 rpm, 30 min, 4°C), then incubated at 4°C with amylose-linked agarose beads (New England Biolabs) for 20 min. Beads were washed with 10 column volumes of ZBA. MBP, MBP-Miz-1-FL, or MBP-Miz-1-ZF was eluted in 3 mL ZBA containing 10 mM maltose, then dialyzed in 2 L ZBA overnight to deplete free maltose using Slide-A-Lyzer dialysis cassettes (Pierce). The resulting protein was concentrated using Amicon Ultra Filter units (Millipore). Purity and yield of the MBP fusion proteins were assessed by SDS-PAGE with Coomassie staining and Bradford Assay (Thermo Fisher Scientific).

### Bind-n-Seq

MBP-Miz-1-FL and MBP-Miz-1-ZF proteins at a range of concentrations (Table 1) were bound to synthetic double-stranded DNA (dsDNA) made from annealed random oligonucleotides with barcodes in BnS binding buffer [0.12 µg/µL Herring Sperm DNA, 100 µM ZnCl_2_, 5 mM DTT, 5% bovine serum albumin] for 30 min with agitation at 25°C. Binding reactions were washed 6×10 min with BnS wash buffer [10 mM Tris (pH 8.5), 100 µM ZnCl_2_, 1 mM MgCl_2_, 5 mM DTT] under different KCl salt concentrations (Table 1). Incubation with EB buffer (Qiagen) containing 10 mM maltose was used to elute bound DNA fragments. To determine the optimal number of amplification cycles for each dsDNA pool, quantitative PCR was performed using the Opticon Monitor system with SYBR green detection (Program: 94°C for 4 min initial denaturation, 26 cycles of 94°C for 30 sec, 63°C for 30 sec, and 72°C for 1 min). DNA was amplified using iProof DNA Polymerase (Bio-Rad, Hercules, CA) and purified using PCR Purification Kit (Qiagen). DNA yield was quantified using a NanoDrop (Thermo Fisher Scientific), and samples were pooled for sequencing. Amplified, pooled samples with barcodes were sequenced using MiSeq (Illumina, San Diego, CA), and reads were sorted and filtered for quality by the MiSeq platform software.

**Table 1:** BnS conditions and enrichment scores.

Barcode	Protein [nM]	Salt [mM]	Highest Fold Enrichment
MBP-Miz-1-FL			
ACA	50	1	17.417
ACC	50	50	20.867
ACG	50	100	14.125
ACT	5	100	9.808
AGA	350	100	11.162
MBP-Miz-1-ZF			
AGC	50	1	25.053
AGG	50	50	26.2
AGT	50	100	16.421
ATA	5	100	19.6
ATC	120	100	10.75

### Motif identification and comparison

*De novo* motif finding was performed as previously described [16]. Sorted, filtered reads were analyzed in randomly sampled clusters of 10,000 reads using MEME. Intermediate motifs were matched back to the original dataset and subsequent rounds of MEME were performed to generate the most enriched motifs for each BnS condition.

To compare the identified motifs to known motifs, the Tomtom motif comparison tool [17] was used to search a database of human and mouse motifs [18] using representative position weight matrices for Mizm1 and Mizm2, using the default significance threshold (E-value <10).

Motif finding in ChIP-seq data sets (GSE48602) was performed using MEME-ChIP with the default parameters (zero or one occurrences per sequence; width  = 6–30 bp). ChIP-seq peak sets were downloaded for two cell types: murine neural progenitor cells (NPCs) and the human mammary epithelial cell line MDA-MB231 (MDA cells). To identify instances of the motif in the Miz-1 ChIP-seq peaks, the position-specific scoring matrices for these motifs were obtained from MEME-ChIP and used as input to FIMO using the default cutoff (p<10^−4^). The motifs obtained from MEME-ChIP were compared to the motifs identified by BnS using Tomtom.

### Electrophoretic Mobility Shift Assay (EMSA)

Probe sequences P1 and P2 were selected from the BnS sequencing reads from MBP-Miz-1-FL and MBP-Miz-1-ZF, respectively. For each probe, we selected a single sequencing read containing the entire consensus sequence from the experimental conditions that yielded the highest enrichment levels (barcodes ACC and AGG). Control probe CP was designed using the Random DNA Sequence generator (http://www.bioinformatics.org/sms2/random_dna.html). Probes (P1, P2, and CP) labeled with 5′ IRDye 700 (Integrated DNA Technologies) were annealed and used for EMSA. Binding reactions also included 100 ng/µl poly(deoxyinosinic-deoxycytidylic) acid nonspecific competitor (Sigma-Aldrich) in ZBA and were performed for 15 min at room temperature. For competitive binding experiments, annealed unlabeled probes were added to the binding reaction prior to addition of labeled probe. Binding reactions were separated on 5% acrylamide gels in 0.5X Tris-borate buffer at 300 V for 60–90 min at 4°C. After electrophoresis, acrylamide gels were visualized using the Odyssey CLx imaging system (Licor), and results were quantified when applicable using Licor ImageStudio software.

### *In vitro* transcription/translation (IVTT)

IVTT was performed using the TNT Quick Coupled Transcription/Translation System (Promega) according to the manufacturer's instructions, using pCS2-hMiz vector or pCS2 empty vector as a control. The reaction mix was supplemented with 1 mM additional alanine and glutamic acid to enhance the yield by providing sufficient quantities of the most frequent amino acids in the sequence of Miz-1.

### Nuclear extracts

293T cells were cultivated in DMEM with 10% fetal bovine serum (FBS). Cells were transfected with pCMV-SPORT6-CUX1 (Thermo Scientific) or empty vector using X-tremeGENE HP (Roche). Two-to-three days after transfection, cells were harvested for nuclear extracts by scraping in PBS. Cells were incubated for 10 min in swelling buffer (10 mM HEPES pH 8, 1.5 mM MgCl2, 10 mM KCl, 0.5 mM DTT, and protease inhibitors), then nuclei were harvested in the same buffer plus 0.05% IGEPAL CA-630. Nuclei were lysed by sonication in ZBA with 10% glycerol to generate nuclear extracts.

### Western blotting

Total protein was isolated using RIPA buffer and separated on 6–12% Bis-Tris gels (Invitrogen), then transferred to PVDF membrane and blocked with 5% non-fat dry milk. Anti-Miz-1 (1∶500; sc22837, Santa Cruz Biotechnology) was applied overnight at 4°C. Where applicable, blots were then re-probed with anti-beta-Actin (1∶10,000; A1978, Sigma-Aldrich). Images were quantified using ImageJ software.

### Luciferase reporter assay

Luciferase reporter vectors were cloned starting with pGL3-Enhancer vector (Promega). By chance, the pGL3-Enhancer vector backbone initially contained the sequence “ATCGAT” upstream of the transcription start site; to produce the pGL3ec control vector containing no potential Miz-1 binding motifs, this sequence was removed by digestion with KpnI and BsgI, blunting, and ligation. To produce vectors containing various putative Miz-1 binding motifs, four repeats of the given motif were inserted upstream of the luciferase gene between the KpnI and XhoI restriction sites. For luciferase assays, HeLa cells were cultivated in DMEM with 10% FBS. Cells were transfected using X-tremeGENE HP with plasmids at a ratio of 20 ng Renilla luciferase, 2 µg pGL3ec or pGL3e-MizM reporter, and 1 µg expression vector [pBabe-Miz-1, pCS2-Miz-1, pCMV-Sport6-Cux1, and/or pBabe empty vector]. To produce varied degrees of Miz-1 overexpression, two different Miz-1 expression vectors were used (pCS2, producing higher expression, and pBabe, producing lower expression), and these vectors were diluted with empty vector in varied ratios to produce different dosages of Miz-1. Overexpression of human c-Myc was induced by transfection with pRc/CMV-c-Myc. Overexpression of Miz-1 and c-Myc was quantified by Western blot. Luciferase assays were performed in 96-well plates according to the manufacturer's instructions using the Dual-Luciferase Reporter Assay (Promega), and results were quantified using a MicroBeta Luminescence Counter (Perkin Elmer). Luciferase values were normalized to Renilla luciferase.

### Statistical analysis

EMSA and luciferase experiments were performed at least in biological triplicate. Numerical data are reported as means ± standard deviations, and statistical significance was determined using the Student's t-test in Microsoft Excel (except where otherwise noted in the text), with a cutoff of *p*<0.05 considered significant. Statistical significance of motif similarity was computed by Tomtom (motif-motif similarity) or FIMO (motif-sequence similarity). Tomtom calculates E-values based on the likelihood of seeing the observed amount of similarity between two motifs by chance, corrected for multiple comparisons.

## Results

### Miz-1 expression and purification

**Figure 1:**
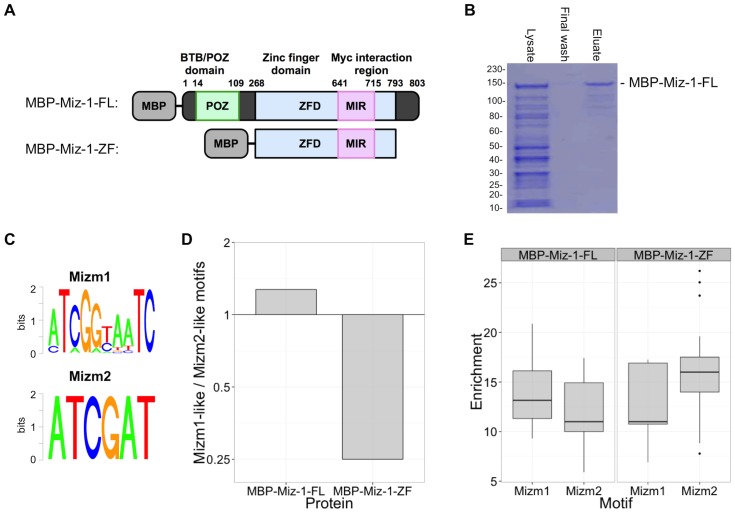
BnS identifies Miz-1 preferred DNA binding motifs. (A) Structure of full length (MBP-Miz-1-FL) and zinc finger domain (MBP-Miz-1-ZF) fusion proteins. MBP-Miz-1-ZF retains the Myc interacting region but not the BTB/POZ domain. (B) Robust expression of purified recombinant MBP tagged proteins was observed at the expected ∼130 kDa size; purification of MBP-Miz-1-FL is shown. Molecular weight standards are labeled in kDa. (C) BnS was performed using MBP tagged proteins, yielding two main motifs, Mizm1 and Mizm2. (D) Ratio of Mizm1-like to Mizm2-like motifs occurring in the list of top 25 BnS hits. (E) Box plot of enrichment scores for Mizm1-like and Mizm2-like motifs identified by BnS.

We produced recombinant human Miz-1 full-length and zinc finger domains (Miz-1-FL and Miz-1-ZF, respectively) using an MBP tag for efficient purification by amylose-linked agarose beads and subsequent elution with maltose (Figure 1A). SDS-PAGE and Bradford Assay confirmed MBP-Miz-1-FL and -ZF purity and concentrations of greater than 2 µM, important for subsequent implementation in the *in vitro* DNA binding assays (Figure 1B and data not shown).

### *De novo* motif identification

We identified Miz-1 binding motifs using BnS, a high-throughput, *in vitro* DNA binding assay that allows for the systematic and rapid detection of DNA binding motifs in parallel. Short, randomly generated oligonucleotides (21 bp binding region) with barcodes were used to generate double stranded DNA fragments that were then bound to MBP-protein constructs and amylose-linked agarose beads, washed and eluted with maltose and identified by massively parallel sequencing to generate approximately 100,000 reads per sample [16]. In this study, MBP-Miz-1-FL and MBP-Miz-1-ZF (including Miz-1 zinc finger residues 269–793) were each analyzed by BnS across five different binding buffer and wash buffer conditions (Table 1). Highly enriched consensus sequence motifs were identified for the full-length (Figure S1) and zinc-finger (Figure S2) constructs. These motifs had significant enrichment of greater than 5-fold and up to 25-fold over background, with hundreds of matching kmers identified in most binding conditions. For both the full-length and zinc-finger proteins, the highest enrichment was observed at conditions of moderate protein concentration and moderate washing stringency (50 mM salt concentration).

Across all binding conditions, every enriched motif had a consensus sequence similar to either “ATCGGTAATC” or “ATCGAT”, so we designated these motifs Mizm1 and Mizm2, respectively (Figure 1C). The sequence “GATTACCGAT”, found repeatedly in the BnS results, is precisely the reverse complement of Mizm1. Of note, Mizm2 is nearly a subsequence of Mizm1, differing by only one base from the first six consensus bases of Mizm1. When Miz-1-FL was used for BnS, Mizm1 was represented more frequently than Mizm2 in the results (14/25 BnS motif hits contained Mizm1, Figure 1D), while Mizm2 was enriched more frequently than Mizm1 when Miz-1-ZF was used (20/25 BnS motif hits contained Mizm2, Figure 1D). The average enrichment scores for the BnS enriched motifs followed a similar trend, with Mizm1 having a slightly higher average enrichment than Mizm2 in the case of Miz-1-FL, while Mizm2 was slightly more enriched than Mizm1 in the case of Miz-1-ZF (Figure 1E); however, these differences were not statistically significant.

### EMSA validates the binding of Miz-1 to the identified motifs

**Figure 2:**
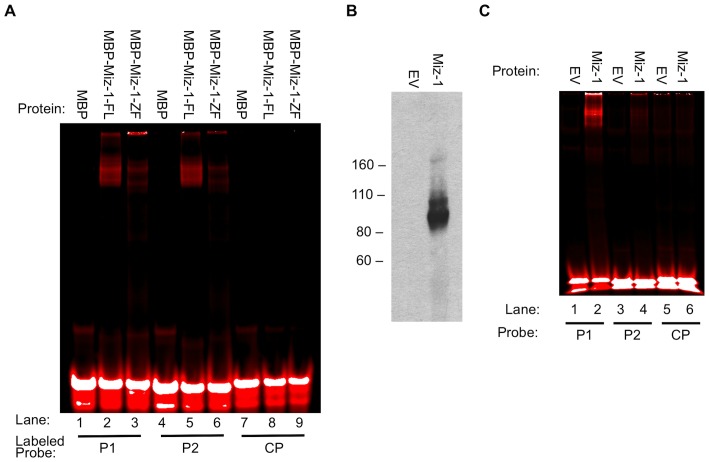
EMSA validates binding of labeled P1 and P2 to full-length Miz-1 and its zinc finger domain, with or without MBP tag. (A) MBP-Miz-1-FL and MBP-Miz-1-ZF bind P1 and P2. MBP alone does not bind either probe, and Miz-1 does not bind the labeled control probe. (B) Untagged Miz-1 (not containing the MBP tag) was produced by IVTT of pCS2-hMiz-1 vector. Molecular weight standards are labeled in kDa. (C) Untagged Miz-1 binds and shifts labeled P1 and P2, but not CP. EV  =  empty vector (IVTT reaction using pCS2 vector backbone).

We employed EMSA to directly assess the affinity of Miz-1 protein for the motifs Mizm1 and Mizm2. Probes were designed by selecting reads from the highest-scoring BnS condition for each protein (Table 2). Probes were selected to contain the consensus sequences Mizm1 (P1) and Mizm2 (P2), using the 21 bp binding region plus the 3 bp barcode that precedes the binding region in the BnS library (ACC for P1, AGG for P2) to generate 24 bp EMSA probes. Note that P2 contains an incomplete second copy of Mizm2, also highlighted. The control probe (CP) was designed using a random sequence generator. Mutant probes (P1m1, P1m2, P2m1, and P2m2) were generated by altering 2–4 of the most conserved bases within the consensus sequence, while an additional mutant probe (P1m3) was designed by selecting a read from the BnS results that contained the same Mizm1 consensus sequence but a different surrounding sequence. Probes labeled with IRDye700 (P1, P2, and CP) were used to allow infrared detection. P1 and P2 were bound and shifted in the gel in the presence of MBP-Miz-1-FL or MBP-Miz-1-ZF (Figure 2A, lanes 2, 3, 5, and 6), while CP was not bound (lanes 8 and 9). Unfused control MBP alone failed to shift any of the probes (lanes 1, 4, and 7), indicating that the observed effect is due to Miz-1 itself and not due to the MBP fusion. Of note, Miz-1-ZF is missing the POZ domain required for Miz-1 homodimerization [19], [20], suggesting that dimerization may not be strictly required for binding to Mizm1 and Mizm2. Figure 2A utilized bacterially expressed Miz-1 with an MBP tag, which could affect DNA binding specificity. In order to address this and further validate the specific binding of Miz-1 to Mizm1 and Mizm2, we used IVTT to produce wildtype untagged Miz-1 protein, which was validated by Western blot (Figure 2B). Untagged Miz-1 protein bound both P1 and P2 in EMSA assays, but did not bind CP (Figure 2C). The relative preference of Miz-1 for P1 vs. P2 was altered by the presence of the MBP tag: MBP-tagged Miz-1 binds roughly equally to P1 and P2 (Figure 2A), while untagged Miz-1 shows a stronger preference for P1 and a lower affinity for P2 (Figure 2C).

**Table 2:** Probe sequences used in EMSA experiments.

Probe	Sequence
P1:	GA ATT ATC GGT AAT CCA TCG AGG T
P1m1:	GA ATT A**GG** **A**GT AA**A** CCA TCG AGG T
P1m2:	GA ATT ATC **C**GT AAT **G**CA TCG AGG T
P1m3:	AC CCT TAT ATC GGT AAT CGG TAA G
P2:	AGG GTT GGT ATC GAT TAT CGA GTT
P2m1:	AGG GTT GGT ATC **A**AT TAT C**T**A GTT
P2m2:	AGG GTT GGT AT**G** GAT TA**A** CGA GTT
CP:	CAA AAG TGC GGC TGC GTG GTG CAC

The motifs Mizm1 (probe P1) and Mizm2 (probe P2) are underlined, while mutations are bolded.

### Competitive EMSA demonstrates specificity of the binding motifs

**Figure 3:**
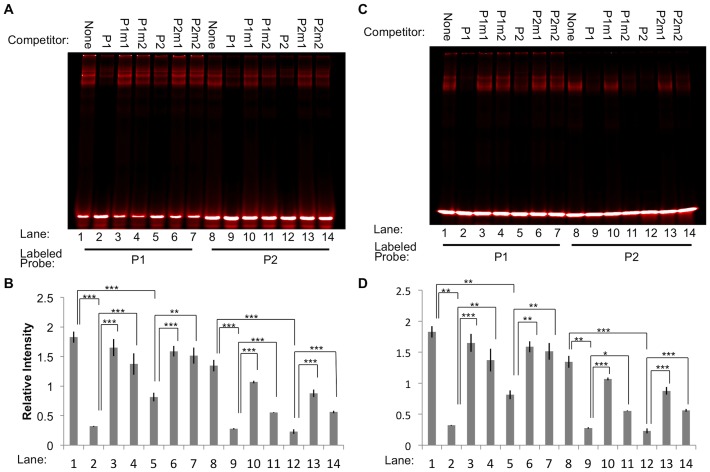
Excess unlabeled probes compete with labeled probes to bind MBP-Miz-1-FL (A-B) or MBP-Miz-1-ZF (C-D). Addition of 200-fold excess unlabeled P1 or P2 abrogates binding of Miz-1-FL or Miz-1-ZF to labeled P1 or P2 (lanes 2 and 5 vs. lane 1; lanes 9 and 12 vs. lane 8). Mutating two to four critical residues in the probe sequences (P1m1, P1m2, P2m1, or P2m2) reduces their ability to compete with labeled probe for binding (lanes 3 and 4 vs. lane 2; lanes 6 and 7 vs. lane 5; lanes 10 and 11 vs. lane 9; lanes 13 and 14 vs. lane 12). Representative images are shown (A, C), along with quantification of three replicate experiments (B, D). * *p*<0.05, ** *p*<0.01, *** *p*<0.001.

To investigate the specificity of the interaction between Miz-1 and its binding motifs, we added various unlabeled probes as competitors to EMSA binding reactions before adding labeled probe. The probe-protein complex was effectively disrupted by addition of 200-fold excess of unlabeled matched probe in the binding reactions (Figure 3, lane 1 vs. 2 and lane 8 vs. 12 in each panel, *p*<0.001). Unlabeled P1 and P2 both effectively out-competed labeled P1 or P2 in complex formation with MBP-Miz-1-FL (Figure 3A-B) or MBP-Miz-1-ZF (Figure 3C-D). However, the extent of competition in any one given condition depended on which labeled and unlabeled probes were used. For example, in the context of MBP-Miz-1-FL (Figure 3B), mixing unlabeled P1 with labeled P1, unlabeled P2 with labeled P2, and unlabeled P1 with labeled P2 reduced the amount of bound labeled probe by 5.3, 6.4, and 4.8 fold, respectively (lanes 2, 9, and 12 vs. lane 1). However, mixing unlabeled P2 with labeled P1 resulted in only a 2.0-fold reduction in binding of the labeled probe (lane 5 vs. lane 1). We observed the same trend when the binding reaction contained MBP-Miz-1-ZF rather than MBP-Miz-1-FL: unlabeled P2 was less able to compete with labeled P1 (lane 5 vs. lane 1) compared to the three other combinations of labeled and unlabeled probes (lanes 2, 9, and 12 vs. lane 1). These data suggest that the affinity of P1 for Miz-1 may be greater than that of P2.

**Figure 4:**
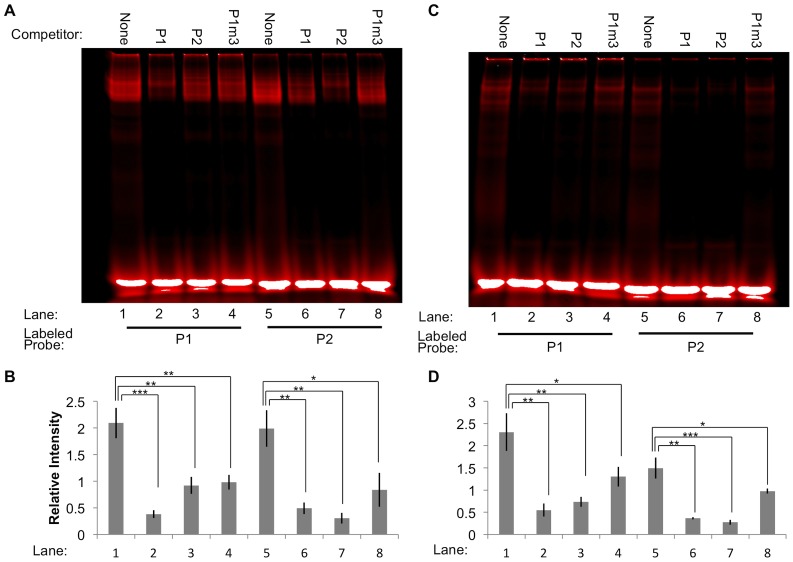
The sequence surrounding the core motif Mizm1 is dispensable for Miz-1 binding. When the sequence surrounding the Mizm1 motif is mutated (P1m3), the unlabeled probe retains its ability to compete with labeled probe P1 or P2 to bind MBP-Miz-1-FL (A-B) or MBP-Miz-1-ZF (C-D). Representative images are shown (A, C), along with quantification of three replicate experiments (B, D). * *p*<0.05, ** *p*<0.01, *** *p*<0.001.

Mutant unlabeled probes were generated containing two-to-four alterations in highly conserved bases from the motif sequences. When these were used in competitive EMSA binding reactions, their ability to block binding of Miz-1 to the labeled probe was significantly attenuated (*p*<0.05 compared to un-mutated competitor). One example is the binding of MBP-Miz-1-ZF to labeled P1, with unlabeled P1, P1m1, or P1m2 as competitors (Figure 3C-D, lanes 1–4). The addition of unlabeled P1 to the reaction (lane 2) caused an 87% reduction in binding of MBP-Miz-1-ZF to labeled P1 as determined by the relative intensity of the shifted bands, but mutating four or two bases of P1 to generate P1m1 and P1m2 competitors (lanes 3 and 4) nearly eliminated the ability of the unlabeled probe to compete for binding (7% reduction for P1m1, 24% reduction for P1m2, compared to no competitor). By comparison, an additional mutant probe, P1m3, was generated that contains Mizm1 with an entirely different surrounding sequence. This probe was still able to efficiently compete with P1 and P2 in binding both MBP-Miz-1-FL and MBP-Miz-1-ZF (35–55% reduction in binding compared to no competitor; Figure 4), indicating that the motif sequence itself is sufficient for effective Miz-1 binding.

### Luciferase reporter assays demonstrate a positive effect of Miz-1 on gene expression

**Figure 5:**
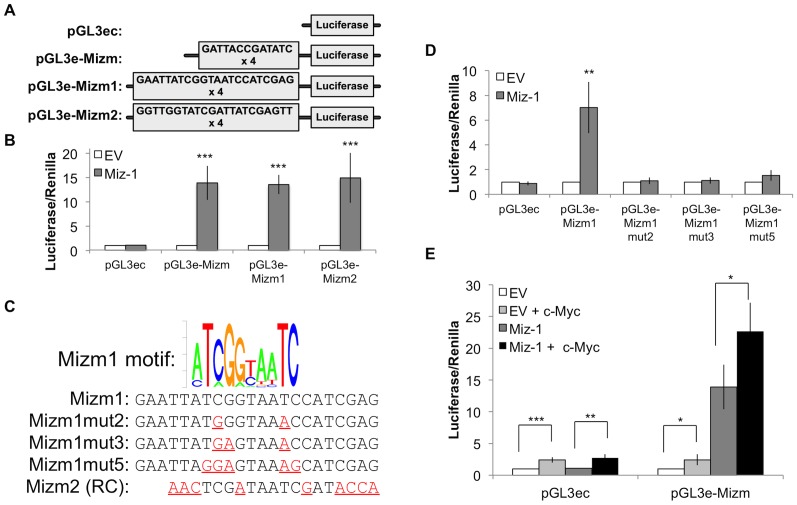
Luciferase reporter assays in HeLa cells demonstrate that Miz-1 activates gene expression via Mizm1. (A) Four luciferase reporter vectors were constructed: pGL3ec containing no putative Miz-1 binding motifs, pGL3e-MizM containing four repeats of both the Mizm1 and Mizm2 motifs upstream of the transcription start site, pGL3e-Mizm1 containing four repeats of the P1 probe sequence, and pGL3e-Mizm2 containing four repeats of the P2 probe sequence. (B) Miz-1 overexpression in HeLa cells produces a statistically significant increase in luciferase reporter activity with all of the three reporter vectors containing putative Miz-1 binding motifs. (C) Three mutant luciferase reporter vectors were constructed, containing two (Mizm1mut2), three (Mizm1mut3), or five (Mizm1mut5) changes in highly conserved bases of the motif. (D) Miz-1 overexpression produces a statistically significant increase in luciferase reporter activation in the presence of Mizm1, but the effect is eliminated by mutating as few as two bases in the motif. (E) Overexpression of c-Myc does not synergize with Miz-1; instead, c-Myc overexpression produces a statistically significant increase in luciferase activity for all conditions: with or without Miz-1 overexpression, and with or without the presence of Miz-1 binding motifs. Luciferase expression was normalized to expression of the Renilla luciferase control reporter vector and to luciferase expression in untreated HeLa cells. * *p*<0.05; ** *p*<0.01; *** *p*<0.001. EV  =  empty vector control; RC  =  reverse complement.

We next examined whether Miz-1 could regulate gene expression by binding to DNA containing the novel consensus motif sequences. We first inserted three different sequences upstream of the luciferase gene in a pGL3-enhancer reporter vector: repeats of both the Mizm1 and Mizm2 consensus motifs with no additional context (pGL3e-Mizm), Mizm1 in the context of P1 (pGL3e-Mizm1), or Mizm2 in the context of P2 (pGL3e-Mizm2; Figure 5A). Each of these reporter constructs was transfected into HeLa cells with or without overexpression of Miz-1 (Figure 5B), revealing a 13 to 15-fold activation of luciferase activity in the presence of any of the three Miz-1 binding motif sequences. Despite the differing affinity of Miz-1 for the two motifs observed by EMSA, Miz-1 is equally capable of inducing transcription through either motif, suggesting that the observed affinity of Miz-1 for P2 is sufficient for productive, transcription-inducing binding *in vivo*.

We predicted that a few specific bases shared by the pGL3e-Mizm, -Mizm1, and -Mizm2 vectors could be critical for Miz-1 binding. To test this hypothesis, we constructed luciferase reporters containing two, three, or five mutations in the Mizm1 sequence (Figure 5C). Luciferase reporter assays demonstrated that mutating as few as two bases in the Mizm1 sequence is sufficient to entirely eliminate the ability of Miz-1 to activate gene expression (Figure 5D).

To examine whether c-Myc might synergize with Miz-1 to activate or repress gene expression in this motif-driven context, given the known ability of the two proteins to bind each other, we conducted luciferase assays in the presence of both proteins. Overexpression of c-Myc produced a small, statistically significant increase in luciferase reporter expression regardless of the presence or absence of Miz-1 or the Miz-1 binding motif (Figure 5E), indicating a general non-specific enhancement of luciferase expression rather than any synergistic effect with Miz-1.

**Figure 6:**
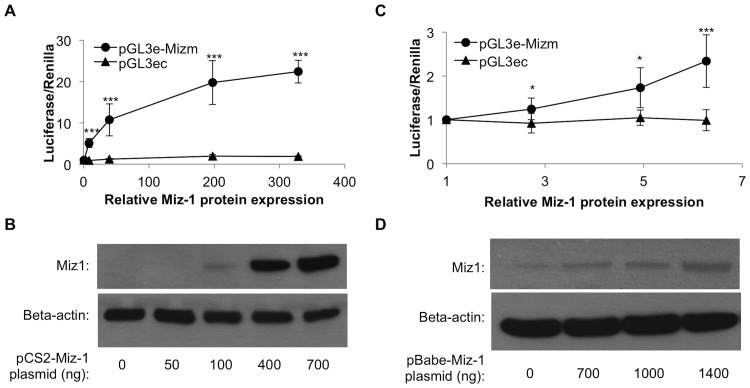
Miz-1 overexpression produces a dose-dependent increase in luciferase reporter expression at high-range (A-B) and low-range (C-D) Miz-1 dosages, while luciferase expression from pGL3ec vector is unaffected by Miz-1. Miz-1 relative protein expression (x-axis in A and C) was determined by quantification of Western blots (representative images shown in B and D) using Image J, and was normalized to beta-actin and to expression in control untransfected HeLa cells. * *p*<0.05; ** *p*<0.01; *** *p*<0.001.

We also investigated the dose-dependence of the transcriptional activation. The pGL3e-Mizm reporter vector was transfected into HeLa cells along with a range of ratios of Miz-1 expression vector to empty vector, in order to generate varied doses of Miz-1 overexpression. A statistically significant, dose-dependent increase in reporter expression was observed with levels of Miz-1 protein overexpression ranging from ∼5-fold to over 300-fold (Figure 6A-B). These effects on gene expression were dependent on presence of the motif sequence; expression of luciferase from the pGL3ec vector lacking the motif sequences was unaffected by addition of Miz-1 (Figure 6A). In separate experiments, we also examined reporter expression in the context of lower relative overexpression of Miz-1 (2- to 6-fold overexpression as determined by Western blot), which may be a more physiologically relevant amount of Miz-1 expression (Figure 6C-D). In this mild overexpression context, a statistically significant increase in reporter expression was also evident at levels of Miz-1 expression less than 3-fold higher than that found in untreated HeLa cells (*p*<0.05).

### Miz-1 binding motifs resemble CUT homeodomain motifs

**Figure 7:**
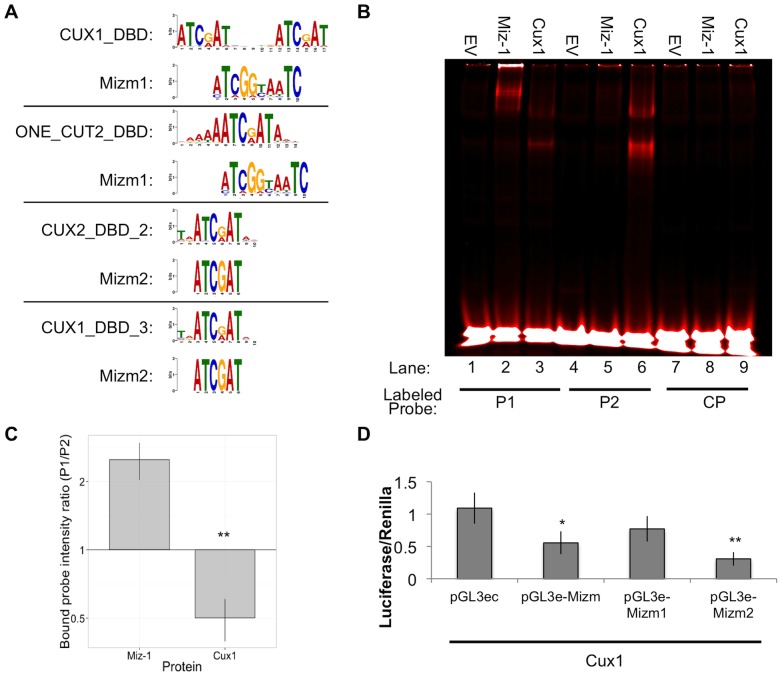
Cux1 binds a similar, but not identical, motif to that identified for Miz-1. (A) Tomtom was used to identify motifs similar to Mizm1 and Mizm2. Alignments are shown between known Cut homeodomain binding motifs and Mizm1 or Mizm2. (B) EMSA reveals that Miz-1 preferentially binds Mizm1 (lane 2 vs. lane 5), while Cux1 preferentially binds Mizm2 (lane 3 vs. lane 6). One representative image is shown (B), along with quantification of three replicate experiments (C). Relative binding intensity of Miz-1 and Cux1 to P1 versus P2 in (C) is defined as the intensity of bound probe in lane 2/lane 5 for Miz-1, and lane 3/lane 6 for Cux1. (D) Cux1-overexpressing HeLa cells show a decrease in luciferase activity when the core Cux1 binding motif (ATCGAT) is present in the reporter vector, but not when the reporter vector contains Mizm1, which does not contain ATCGAT. * *p*<0.05; ** *p*<0.01; *** *p*<0.001.

Position weight matrices for Mizm1 and Mizm2 (Figure 1C) were used as input into the Tomtom motif comparison tool in order to identify any known motifs with similarity to the Miz-1 motifs that we characterized earlier, using a database of human and mouse transcription factor binding sites. Tomtom identified 14 motifs significantly similar to Mizm1 and 14 motifs similar to Mizm2 (Table 3; E-value <10). A strikingly high number of transcription factors containing the CUT homeodomain were represented in the Tomtom results: 29% of the matches for Mizm1 (4 of 14) and 64% of the matches for Mizm2 (9 of 14) were members of the CUT homeodomain family. Most CUT homeodomain proteins have the consensus sequence ATCGAT as the core of their binding motif, which aligns with the Miz-1 binding motifs we have identified (Figure 7A).

**Table 3:** Motifs with significant similarity to Mizm1 and Mizm2 identified using Tomtom.

Mizm1		Mizm2	
Matching motif	p-value	Matching motif	p-value
ERG_full_2	0.0027	**CUX2_DBD**	**0.0003**
FLI1_full_2	0.0031	**CUX2_DBD_2**	**0.0004**
ERG_DBD_2	0.0034	PAX7_full	0.0005
**CUX1_DBD**	**0.0034**	**CUX1_DBD_3**	**0.0006**
VENTX_DBD_2	0.0035	PAX3_DBD	0.0009
FLI1_DBD_2	0.004	**CUX1_DBD_2**	**0.0009**
HESX1_DBD_2	0.0048	**CUX1_DBD**	**0.001**
LHX9_DBD_2	0.0083	**ONECUT1_full**	**0.001**
**ONECUT2_DBD**	**0.0089**	**ONECUT2_DBD**	**0.001**
FLI1_DBD	0.0093	PAX7_DBD	0.0014
**CUX2_DBD**	**0.0098**	**ONECUT1_DBD**	**0.0019**
**ONECUT1_full**	**0.0105**	**ONECUT3_DBD**	**0.0052**
ERG_DBD	0.0119	VENTX_DBD	0.0074
IRF7_DBD_2	0.0126	IRF7_DBD_2	0.008

Cut-homeodomain family members are in bold.

### Miz-1 and Cux1 have differential preferences for Mizm1 and Mizm2

We hypothesized that if Miz-1 and CUT homeodomain proteins bind similar motifs, they may compete to bind the same DNA sequences in cells and have some unknown inter-related function. To begin to test this hypothesis, we focused on the most well-studied member of the CUT homeodomain protein family, Cux1, which binds the consensus sequence ATCGAT [21]. To determine whether Cux1 is likely to compete with Miz-1 to bind the same sequences, we produced nuclear extracts from 293 T cells transfected with Cux1 or empty vector as a control. Multiple Cux1 bands including the p200 and p110 isoforms were detected by Western blot (data not shown), in accordance with previous studies indicating that Cux1 is proteolytically processed to generate multiple different isoforms with higher DNA-binding affinity [22], [23]. When these nuclear extracts were used in EMSA assays, both Miz-1 and Cux1 bound P1 and P2 but not CP (Figure 7B). We observed that Miz-1 bound P1 more strongly than P2, while Cux1 bound P2 more strongly than P1. Quantification of EMSA bands validated this observation (Figure 7C): the intensity of the probe shifted by Miz-1 was approximately 2.5-fold higher for P1 than for P2, while the intensity of the probe shifted by Cux1 was approximately 2-fold higher for P2 than for P1. In agreement with the concept that Cux1 preferentially binds Mizm2, luciferase assays demonstrated that Cux1 represses luciferase expression from pGL3e-Mizm1 and pGL3-Mizm2, the two reporter constructs containing the sequence “ATCGAT” (Figure 7D). Cux1 did not repress gene expression from pGL3e-Mizm1, which does not contain the sequence “ATCGAT”, suggesting that Cux1 depends on the presence of that specific hexamer sequence for its transcriptional function. These experiments demonstrate that while Miz-1 and Cux1 indeed bind very similar motifs, they each have a unique preferred motif and likely do not directly compete to bind the same genomic motifs *in vivo*.

### Miz-1 ChIP-seq data reveals enrichment of motifs similar to Mizm1 and Mizm2

**Figure 8:**
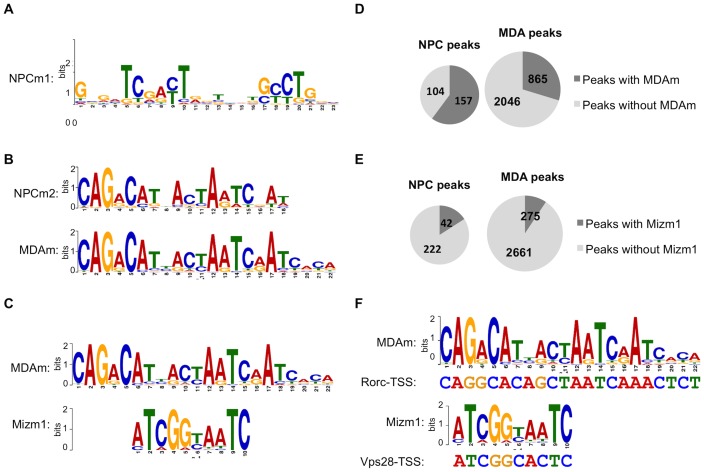
Motifs enriched in Miz-1 ChIP-seq peaks match Mizm1. (A) The top-scoring peak in NPCs, as reported by Wolf et al., has little similarity to Mizm1. (B) Tomtom alignment of the top-scoring motif in MDA peaks (MDAm) with the second-top scoring motif in NPC peaks (NPCm2), demonstrating that they are nearly identical to each other (*p* = 8.8×10^−10^). (C) Tomtom alignment of Mizm1 with the central portion of MDAm, showing statistically significant similarity (*p* = 0.009). (D) FIMO was used to identify ChIP-seq peaks containing instances of MDAm in the NPC (left) and MDA (right) Miz-1 ChIP-seq peak sets, using a cutoff of *p*<0.0001. (E) FIMO was used to identify ChIP-seq peaks containing instances of Mizm1 in the NPC (left) and MDA (right) Miz-1 ChIP-seq peak sets, using a cutoff of *p*<0.0001. (F) FIMO was used to search the ChIP-seq peaks that Wolf et al. validated by ChIP-qPCR for matches to the MDAm and Mizm1 motifs. Examples of statistically significant matches are shown (*p* = 1.7×10^−7^ for MDAm in RORC-TSS; *p* = 1.22×10^−5^ for Mizm1 in Vps28-TSS).

A study from Wolf, et al. was recently published containing global genomic binding profiles for Miz-1 in two cell types, obtained by ChIP-seq [15]. We retrieved the Miz-1 peak data from the NCBI Gene Expression Omnibus and submitted it to MEME-ChIP to identify motifs in the data. Wolf, et al. reported the top motif returned by MEME-ChIP in the murine sample: a long, relatively permissive motif with no resemblance to Mizm1 or Mizm2 that we designate NPCm1 (Figure 8A). However, when we repeated and extended the previous analysis of this data, we found that the second motif returned by MEME-ChIP for the murine NPC data (NPCm2), as well as the top motif returned for the human MDA cell data (MDAm), are also very highly enriched, with E-values of 8.2×10^−29^ and 1.10×10^−195^, respectively. These motifs are quite different from NPCm1 and are nearly identical to each other, as determined by Tomtom (Figure 8B; *p* = 8.8×10^−10^). The central portions of MDAm and NPCm2 are also significantly similar to the motif Mizm1 as determined by Tomtom (Figure 8C; *p* = 0.009).

We used FIMO to identify instances of MDAm in both the NPC and MDA peak sets; MDAm was present in 60.2% of peaks in the mouse data set and 29.7% of peaks in the human data set (Figure 8D). We also used FIMO to identify instances of Mizm1 in the ChIP-seq peak sets (Figure 8E). Mizm1 was present in a lower fraction: 16.1% of peaks in the mouse data and 9.5% of peaks in the human data. The prevalence of MDAm in both peak sets suggests relatively high *in vivo* relevance of this motif; the lower prevalence of Mizm1 suggests that the binding specificity of physiologically expressed Miz-1 in its native genomic context may differ at least somewhat.

An important next step would be to determine whether the ChIP-seq peaks containing these motifs could be validated by ChIP-qPCR. We reasoned that due to the high prevalence of MDAm in the peak sets, some of the peaks that were already validated by Wolf et al. might contain the motifs. Therefore we used the UCSC In Silico PCR tool to identify the sequences amplified by the qPCR primers given in the supplemental material accompanying the paper, and we used FIMO to search for instances of the motifs in the regions that were amplified by ChIP-qPCR. Indeed, FIMO identified sequences matching MDAm in four of the 10 peaks that Wolf et al. validated by ChIP-qPCR, including Rorc (Figure 8F). Similarly, FIMO identified sequences matching Mizm1 in 4 of the 10 peaks, including Vps28 (Figure 8F). This result validates Miz-1 binding to genomic regions containing MDAm and Mizm1.

Intriguingly, we noted that the Rorc1 luciferase reporter vector constructed by Wolf et al. also contains an excellent match to MDAm (p = 6.61×10^−7^, determined by FIMO). This raises the possibility that the active sequence in the Rorc1 luciferase vector could actually be MDAm rather than NPCm. The researchers observed approximately 8–20-fold activation of the reporter by Miz-1, which is similar to the level we observed using reporter vectors that contain Mizm1. Overall, this reporter data along with the strong agreement between the *in vitro* BnS data and the *in vivo* MEME-ChIP data provides robust support for the functional importance of these motifs in directing Miz-1 recruitment to DNA.

### Miz-1 ChIP-seq peaks containing MDAm are predominantly located outside of proximal promoter regions

**Figure 9:**
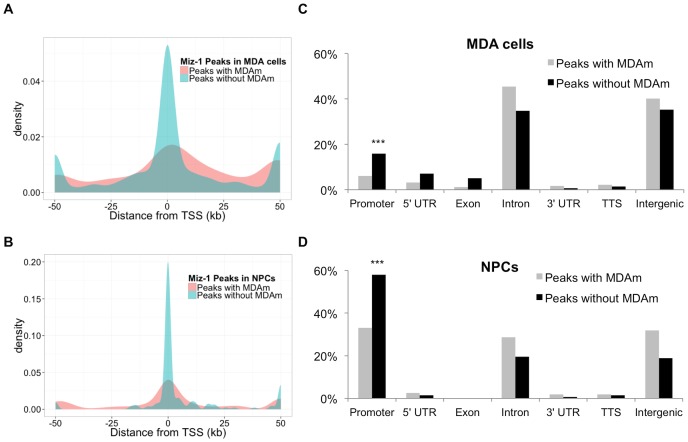
Analysis of ChIP-seq peak locations with respect to genes. (A-B) Peaks lacking MDAm are highly concentrated within 1 kb of the TSS in the ChIP-seq data sets from MDA cells (A) and NPCs (B), while in both cases, peaks containing MDAm are less likely to be localized near the TSS. Density plots were generated in R using the ggplot2 package; peaks occurring more than 50 kb from the nearest TSS were plotted at +/−50 kb. (C-D) Homer annotations of peak locations for ChIP-seq peaks from MDA cells (C) and NPCs (D). The promoter is defined as −1 kb to +100 bp surrounding the TSS; TTS (transcription termination site) is defined as −100 bp to +1 kb surrounding the TTS. Peaks containing MDAm were identified using FIMO, and the distance to nearest TSS and gene-centered annotations were determined using Homer with all RefSeq human (A, C) or mouse (B, D) genes. *** *p*<0.0001 (Chi-squared test).

Miz-1 has previously been reported to bind Inr sequences near transcription start sites (TSS) that do not resemble the motifs we describe in this report. We hypothesized that motif-dependent binding of Miz-1 may represent an Inr-independent form of binding that is less likely to be localized in proximal promoter regions, but rather occurs in gene bodies, enhancers, or other regions. To test this hypothesis, we generated density plots of Miz-1 ChIP-seq peak locations with respect to the nearest TSS (Figure 9A-B). In both NPCs and MDA cells, peaks without motifs are highly concentrated near the TSS, while peaks with MDAm motifs are distributed broadly throughout more distal regions. In the NPC peak set, 36.7% of peaks containing MDAm occur within 1.5 kb of a TSS, while 60% of peaks without MDAm occur in the same interval. Similarly, in the MDA peak set, 14.8% of peaks containing MDAm occur within 1.5 kb of a TSS, while 33.2% of peaks without MDAm occur in the same interval. These differences are statistically significant (*p* = 9.9×10^−5^ for NPC peak set, *p*<2.2×10^−16^ for MDA peak set; Chi-squared test).

We repeated the analysis, this time using Homer's classification of peaks into genome regions rather than distance from TSS as the metric (Figure 9C-D). Again, peaks without motifs were more likely to be found in promoter regions in both cell types (*p*<0.0001; Chi squared test). Peaks containing MDAm were enriched in introns and intergenic regions, which, in this classification scheme, includes genomic features like enhancers.

## Discussion

In this work we have defined and validated a novel preferred DNA binding motif for the zinc finger transcription factor Miz-1 using BnS with both the full length Miz-1-FL and extended zinc finger domain Miz-1-ZF protein constructs. Miz-1-ZF retains some ability to bind Mizm1/Mizm2 despite loss of the N-terminal 268 amino acids of Miz-1, including the BTB/POZ domain, although the level of binding appears slightly lower. This suggests that dimerization through the POZ domain is not strictly required for DNA binding. Instead, the extended zinc finger domain encompassing most of the protein is likely responsible for binding the DNA motif, in accordance with the known DNA binding function of C2H2 zinc fingers. The structures of Miz-1 zinc fingers 5–8 [24] and 8–10 [25] have been solved, and it is also possible to predict the binding specificity of all 13 ZFs from their sequences: 5′-A(T/C)C NAG (G/T)CN NNA N(T/C)A GTC GAT NAA G(T/C)C GAT NNT NTC GA(T/C)-3′ [25]. However, this predicted binding specificity has little obvious similarity to the motifs we identified experimentally, or to the motif identified by Wolf, et al., suggesting that motif prediction from the protein's sequence may not reflect the protein's DNA sequence affinity.

BnS analysis may tend to identify only high affinity DNA motifs, while there may be several motifs for a given protein based on its structural conformation that vary in affinity, but nonetheless are functionally relevant. The consensus motifs identified for Miz-1, Mizm1 and Mizm2, were both highly enriched over background and were further analyzed for their ability to be bound by Miz-1 protein *in vitro*. EMSA analysis confirmed Miz-1 binding to these motifs, and demonstrated that altering as few as two bases is sufficient to disrupt binding of Miz-1 to the motif sequence. The affinity of Miz-1-FL for Mizm2 is much lower than its affinity for Mizm1 when compared by EMSA; however, Miz-1 is equally capable of inducing transcription through the two motifs in luciferase assays. Additionally, the motifs identified by MEME-ChIP are similar to Mizm1 and Mizm2 but longer, suggesting that more of the zinc fingers of Miz-1 may be able to bind DNA *in vivo* as compared to in a BnS assay.

Surprisingly, the recently published Miz-1 binding motif by Wolf, et al. differs completely from the motifs we describe in this report defined by both BnS and MEME-ChIP. Our results do not necessarily contradict the previously reported motif NPCm1; Miz-1 contains thirteen ZF motifs that could each have differing DNA sequence specificities, making it possible that the Miz-1 protein binds multiple independent motifs depending on the context. The mechanism of action of polydactyl ZF proteins such as Miz-1 may be quite different from those that contain only a few ZF and may rely more on protein-protein interactions. In most multi-finger proteins, which can have more than 35 fingers, only 3–5 fingers are usually involved in DNA binding; the others may be involved in RNA or protein binding, and in some cases may even have overlapping functions [26], [27]. An additional potential complication is that motif-driven binding of Miz-1, such as binding to Mizm1 and Mizm2, likely represents only a subset of Miz-1 binding *in vivo*. Miz-1 has numerous binding partners such as Myc that also can themselves bind to DNA or chromatin and in so doing may tether Miz-1 to the genome, which may account for a substantial fraction of its genomic binding, depending on the cell type, and alter DNA sequence recognition.

Understanding the genomic binding of Miz-1 may shed light on cancer and stem cell related gene expression programs co-regulated by Miz-1 and Myc. Miz-1 binds initiator sequences in the core promoters of target genes thereby modulating their expression [1], [13], [28]. The two existing studies of global genomic Miz-1 binding differ substantially in the proportion of Miz-1 binding reported to occur in proximal promoters vs. farther from transcription start sites [9], [15], suggesting possible context-dependent differences in the global DNA binding pattern of Miz-1. Functionally, Miz-1 expression correlates with favorable outcomes in neuroblastoma [6], [29], while excess Myc functions as a potent oncoprotein [30], suggesting a possible antagonistic relationship between Miz-1 and Myc in cancer. However, in some cases Myc and Miz-1 can work in concert to promote tumorigenic functions [31], suggesting that their relationship is much more complex than simple antagonism. We have postulated that Miz-1 may tether its binding partners, including Myc, to the genome through binding to specific DNA motifs such as Mizm1 and Mizm2. However, we did not observe a synergistic effect of c-Myc on Miz-1-driven reporter activity. At least in the cell culture context that we have examined, this suggests that Miz-1 may bind through Mizm1 and Mizm2 independent of Myc to activate transcription.

The Miz-1 binding motifs we identified are strikingly similar to the known transcription factor binding motif for the homeobox protein CDP/Cux1 (TRANSFAC M00104), which includes the consensus sequence ATCGAT [21]. This suggests the possibility that Cux1 and Miz-1 could potentially compete for genomic binding. Cux1 is proteolytically cleaved to form a p110 isoform with stronger DNA binding affinity [21]. In agreement with the idea that proteolytic cleavage is required for strong DNA binding by Cux1, we used IVTT to produce full-length Cux1 for EMSA experiments, but the un-cleaved Cux1 product failed to bind detectable levels of Mizm1 or Mizm2 (data not shown). In contrast, Cux1 produced in 293T cells was cleaved to form multiple smaller isoforms observed by Western blot, and robustly bound Mizm2 in EMSA assays. Overall, the data suggest that despite the sequence similarity between the two proteins' motifs, Miz-1 and Cux1 are unlikely to compete for the same sequences *in vivo* due to their differing preference for Mizm1 vs. Mizm2. However, we cannot at this point rule out the possibility that the two proteins may, in some context, compete to bind the same motif.

In this work, we used BnS to define potential direct DNA binding functions of Miz-1. The novel motifs that we have identified may guide localization of Miz-1 *in vivo*, where it can recruit from its extensive host of binding partners to regulate gene expression and ultimately cell fate. Future work including additional genomics studies will help to further characterize how Miz-1 functions on chromatin.

## Supporting Information

Figure S1 **BnS motifs obtained using MBP-Miz-1-FL protein.** Each of the five barcodes refers to a separate BnS experiment using the stated concentrations of protein and salt. For each barcode, the top five highest scoring motifs are reported.
(PDF)

Figure S2 **BnS motifs obtained using MBP-Miz-1-ZF protein.** Each of the five barcodes refers to a separate BnS experiment using the stated concentrations of protein and salt. For each barcode, the top five highest scoring motifs are reported.
(PDF)
