# Correction: Miz-1 activates gene expression via a novel consensus DNA binding motif

**DOI:** 10.1371/journal.pone.0108565

**Published:** 2014-09-16

**Authors:** The PLOS ONE Editors

After publication of the manuscript, Wolf, Eilers and colleagues pointed out that the two motifs identified in this analysis and in particular Mizm2, have homology to the extended sequence motif published by Wolf et al. Nat Commun (2013) 4:2535. Further, the VPS28 motif shown in Figure 8F is identical to the Miz1 binding site shown by Wolf et al. in Figure 3e of their paper. We regret this oversight. From the Wolf et al. (2013) paper and ours here, therefore, we conclude that two independent experimental approaches identified highly similar and overlapping binding motifs for Miz1.
