# Supplementary material for: Miz-1 Activates Gene Expression via a Novel Consensus DNA Binding Motif
Source: PLoS One. 2014 Jul 1;9(7):e101151. doi: 10.1371/journal.pone.0101151 (PMC4077741; doi:10.1371/journal.pone.0101151)

Supplementary Figure 1. BnS motifs obtained using MBP-Miz-1-FL.

Barcode ACA: 50 nM MBP-Miz1-FL, 1 mM salt

BC FG BG Enrichment Forward motif | Reverse comp. motif

ACA 208 11 17.417

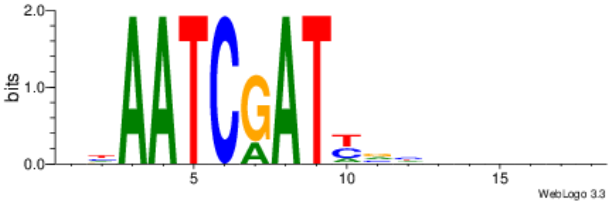

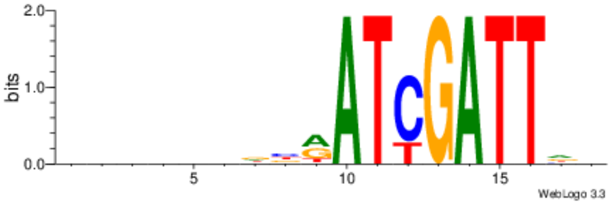

ACA 349 20 16.667

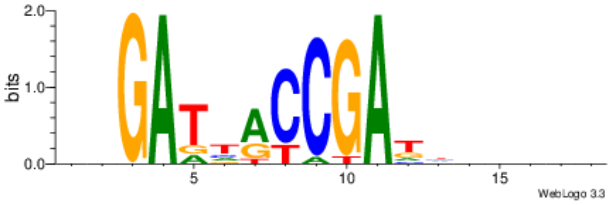

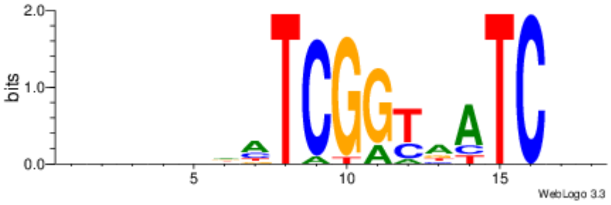

ACA 57 3 14.500

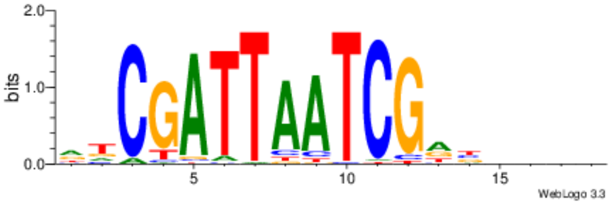

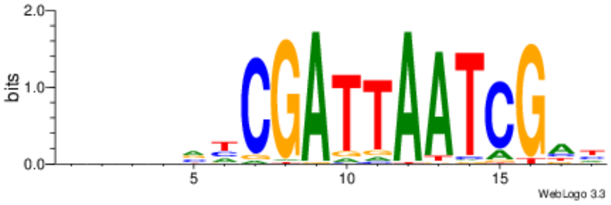

ACA 228 15 14.312

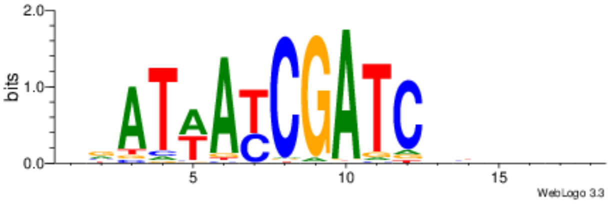

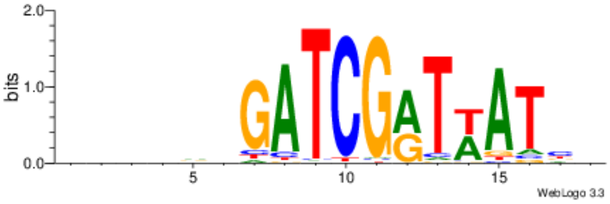

ACA 67 4 13.600

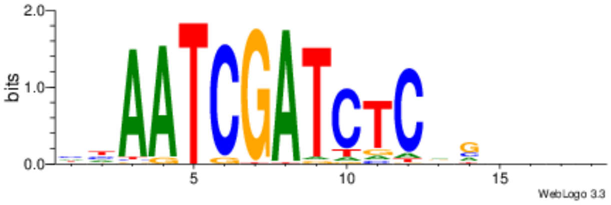

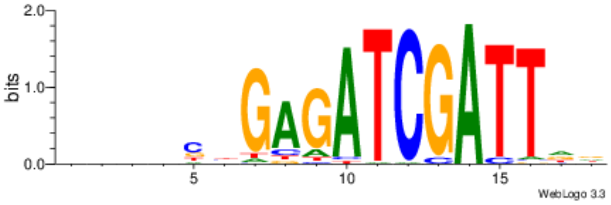

Barcode ACC: 50 nM MBP-Miz1-FL, 50 mM salt

BC FG BG Enrichment Forward motif | Reverse comp. motif

ACC 312 14 20.867

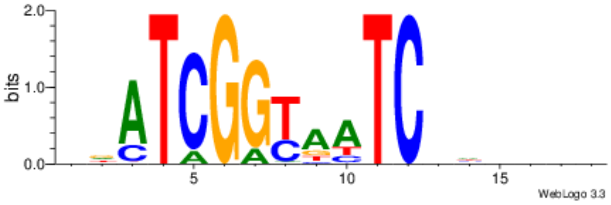

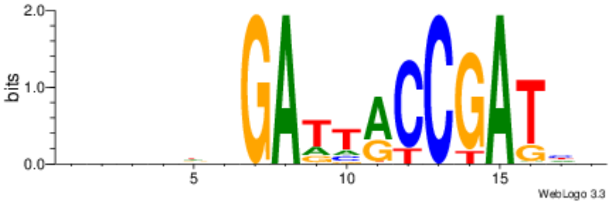

ACC 559 28 19.310

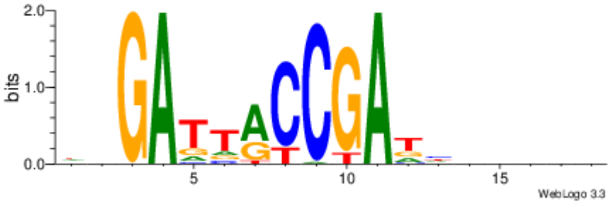

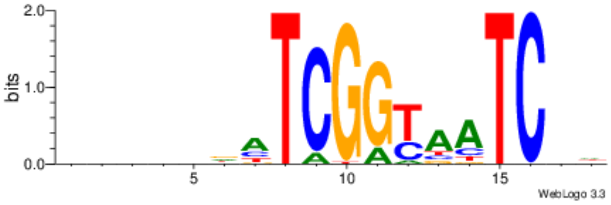

ACC 279 15 17.500

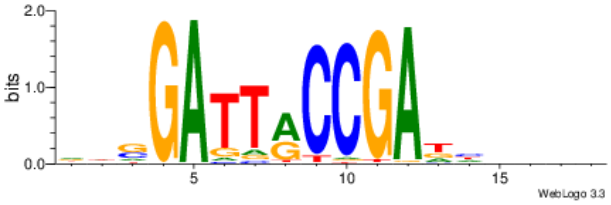

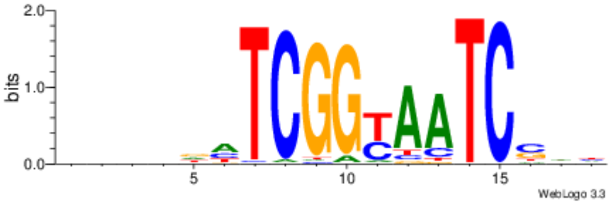

ACC 355 20 16.952

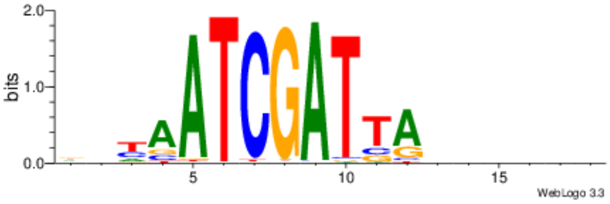

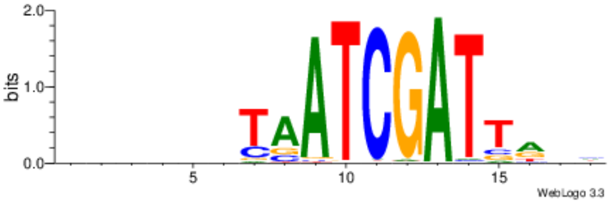

ACC 263 16 15.529

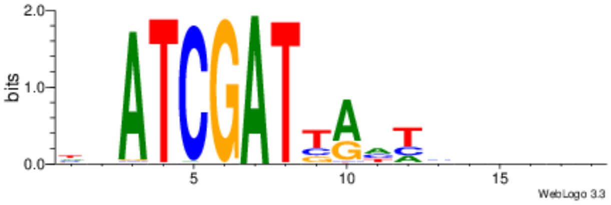

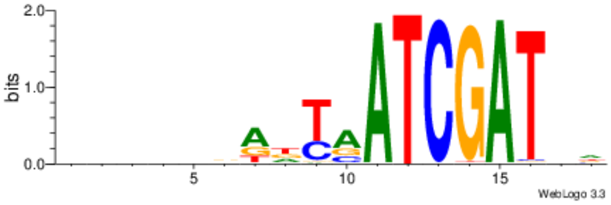

Barcode ACG: 50 nM MBP-Miz1-FL, 100 mM salt

BC FG BG Enrichment Forward motif Reverse comp. motif

ACG 112 7 14.125

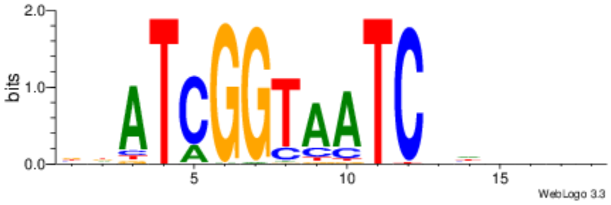

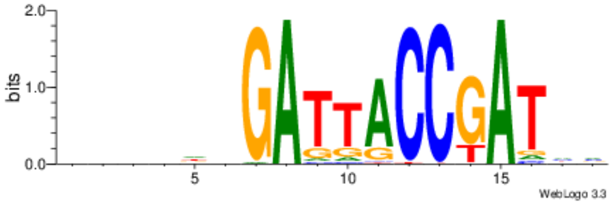

ACG 309 22 13.478

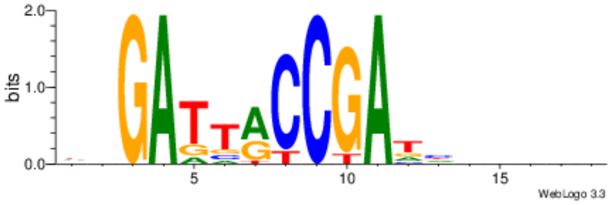

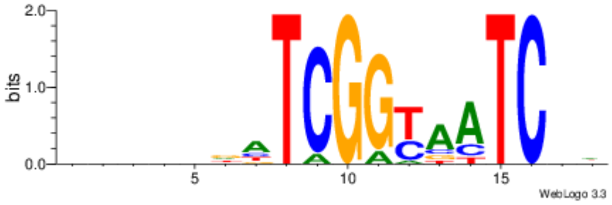

ACG 63 4 12.800

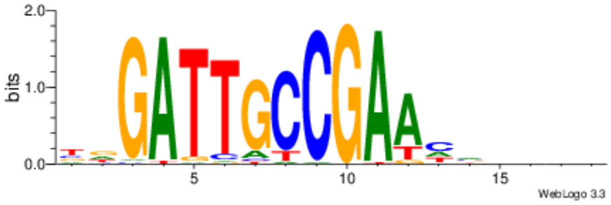

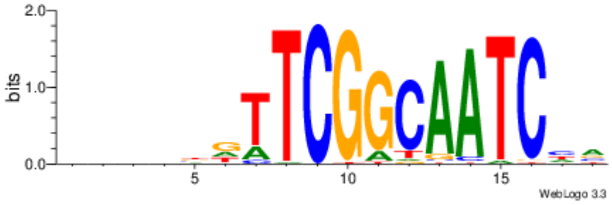

ACG 176 4 11.800

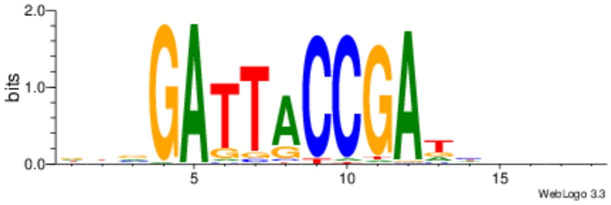

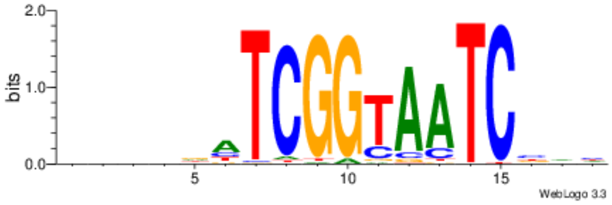

ACG 139 11 11.667

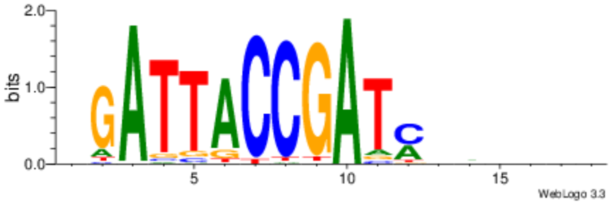

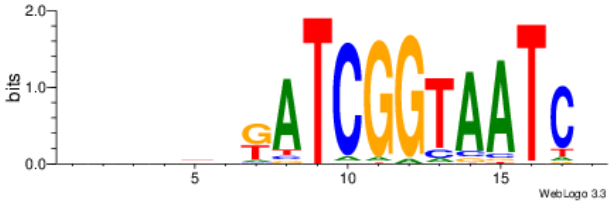

Barcode ACT: 5 nM MBP-Miz1-FL, 100 mM salt

BC FG BG Enrichment Forward motif Reverse comp. motif

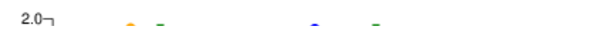

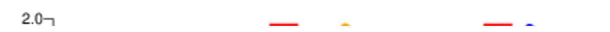

ACT 254 25 9.808

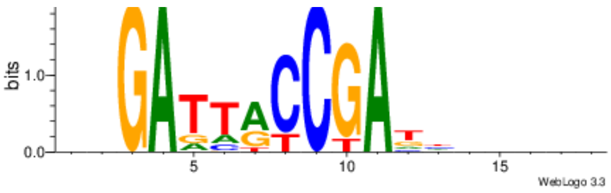

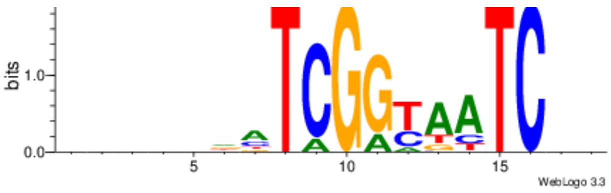

ACT 95 9 9.600

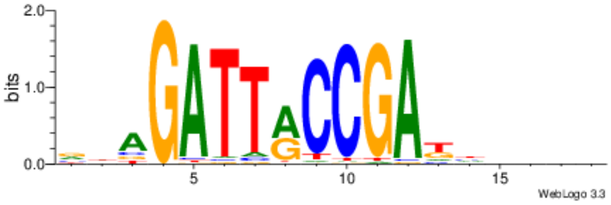

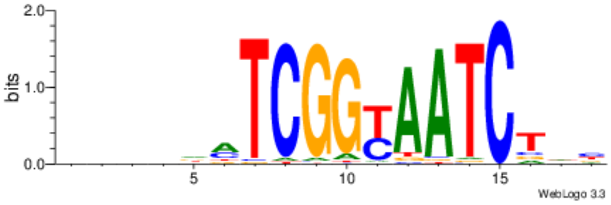

ACT 149 15 9.375

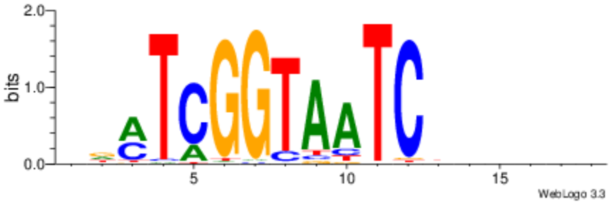

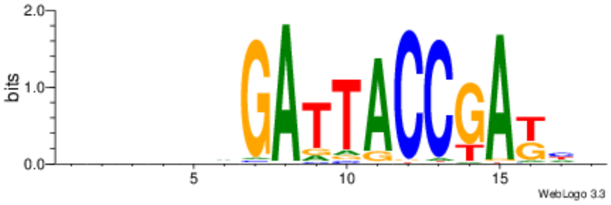

ACT 265 41 6.333

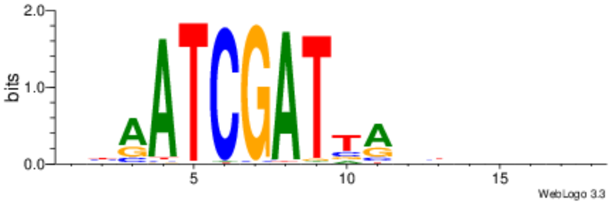

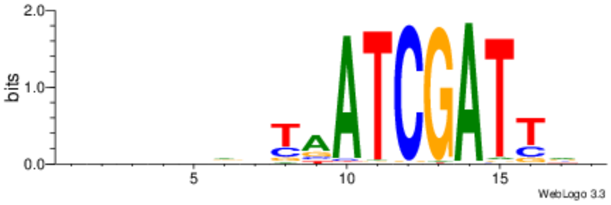

ACT 181 30 5.871

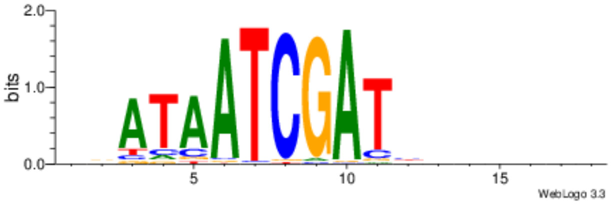

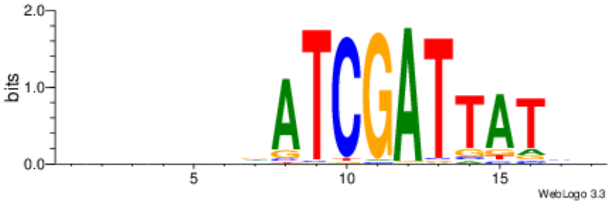

Barcode AGA: 350 nM MBP-Miz1-FL, 100 mM salt

BC FG BG Enrichment Forward motif — Reverse comp. motif

AGA 412 36 11.162

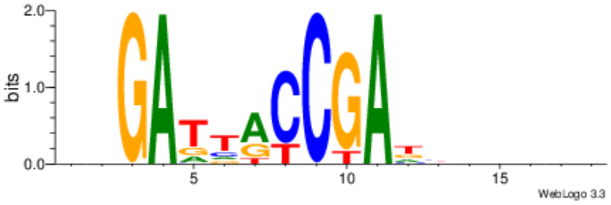

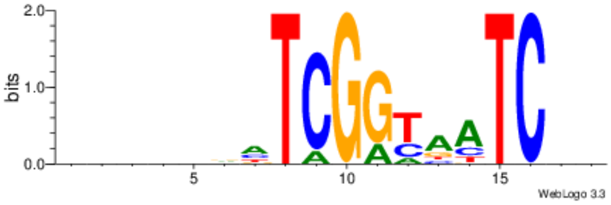

AGA 385 34 11.029

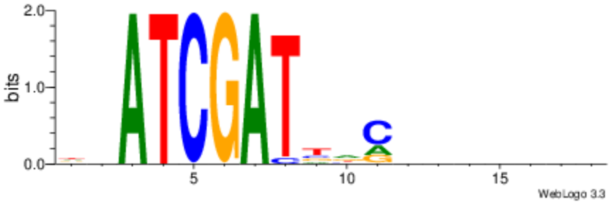

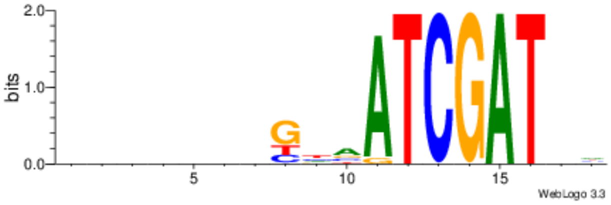

AGA 494 44 11.000

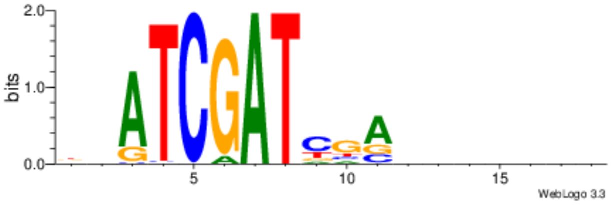

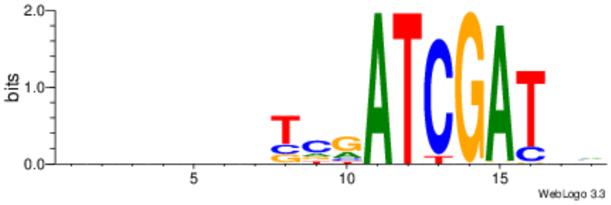

AGA 388 36 10.514

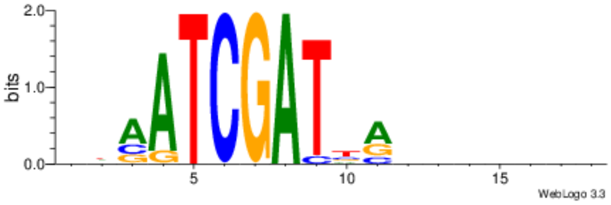

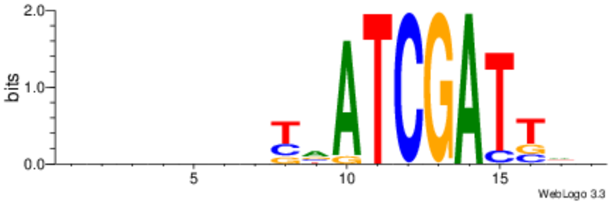

AGA 179 18 9.474

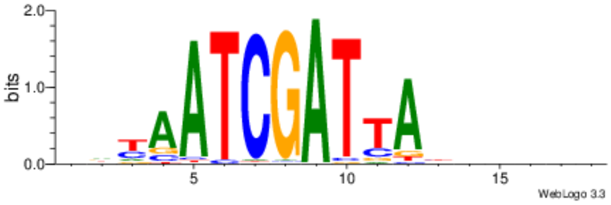

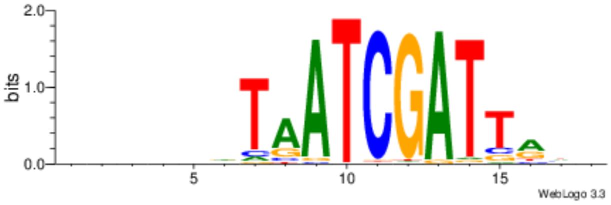

Supplement: Figure S1 — BnS motifs obtained using MBP-Miz-1-FL protein. Each of the five barcodes refers to a separate BnS experiment using the stated concentrations of protein and salt. For each barcode, the top five highest scoring motifs are reported. (PDF) [file pone.0101151.s001.pdf]
