# Supplementary material for: Miz-1 Activates Gene Expression via a Novel Consensus DNA Binding Motif
Source: PLoS One. 2014 Jul 1;9(7):e101151. doi: 10.1371/journal.pone.0101151 (PMC4077741; doi:10.1371/journal.pone.0101151)

Supplementary Figure 2. BnS motifs obtained using MBP-Miz-1-ZF.

Barcode AGC: 50 nM MBP-Miz1-ZF, 1 mM salt

BC	FG	BG	Enrichment	Forward motif	Reverse comp. motif
AGC	475	18	25.035	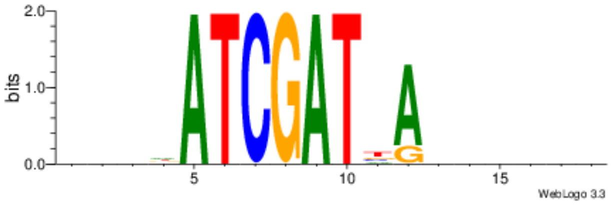	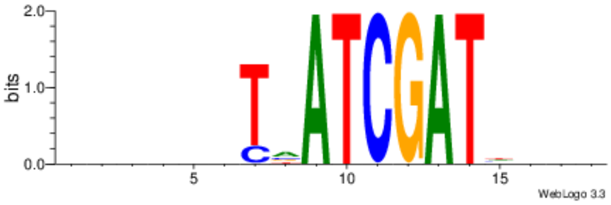
AGC	734	30	23.710	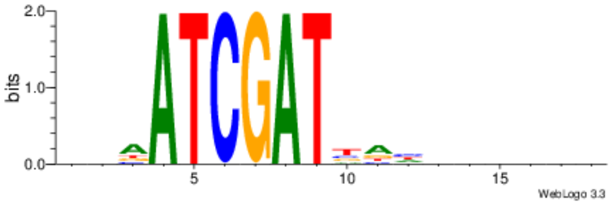	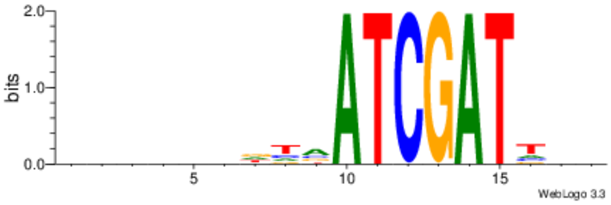
AGC	352	17	19.611	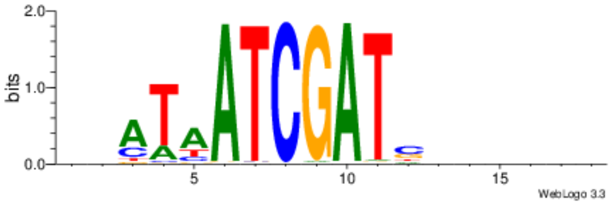	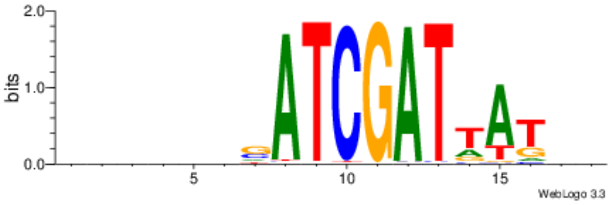
AGC	371	21	16.909	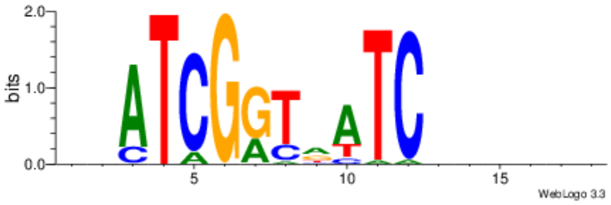	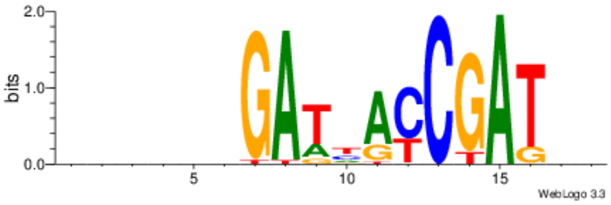
AGC	705	41	16.810	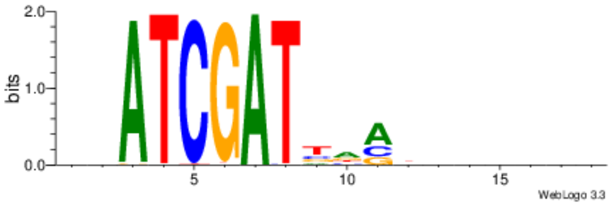	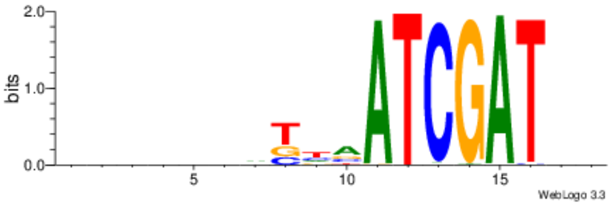

Barcode AGG: 50 nM MBP-Miz1-ZF, 50 mM salt

BC	FG	BG	Enrichment	Forward motif	Reverse comp. motif
AGG	261	9	26.200	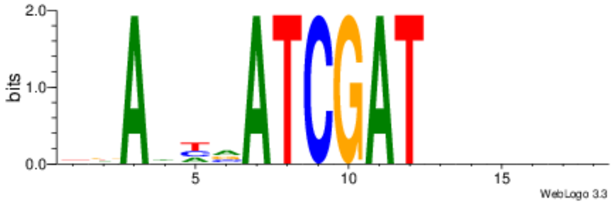	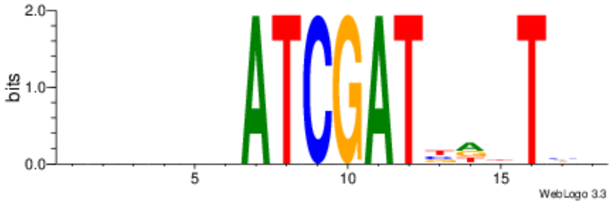
AGG	258	14	17.267	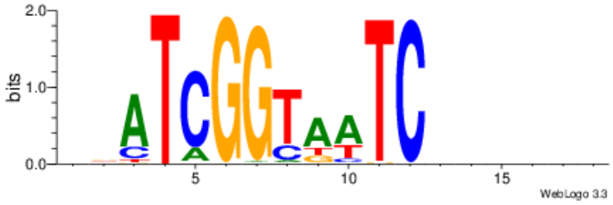	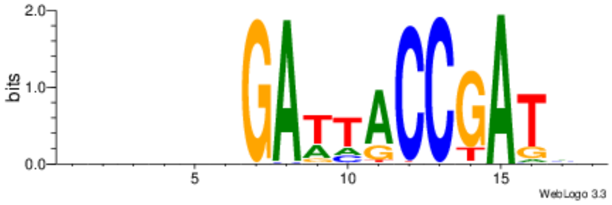
AGG	304	18	16.053	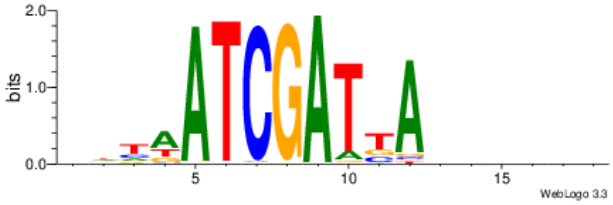	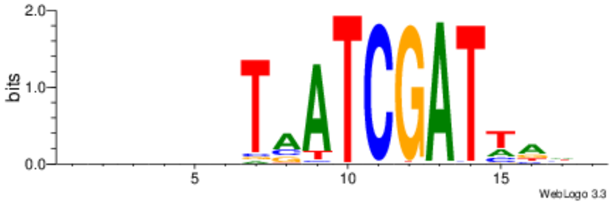

AGG 335 20   16.000

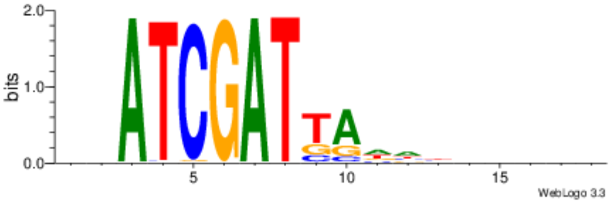
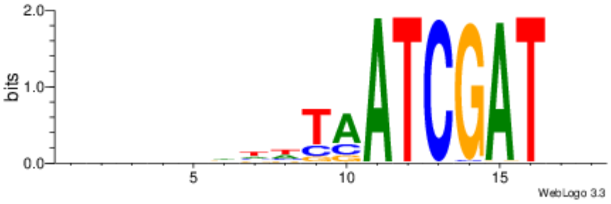

AGG 354 24   14.200

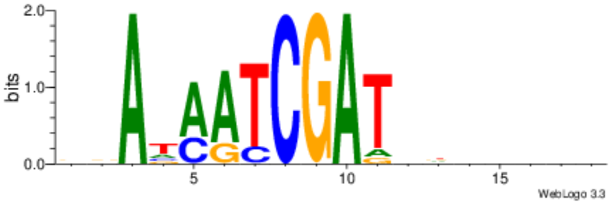
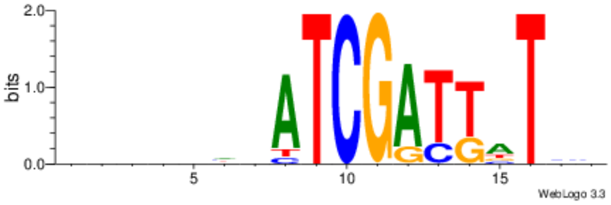

Barcode AGT: 50 nM MBP-Miz1-ZF, 100 mM salt

BC   FG BG Enrichment Forward motif   Reverse comp. motif

AGT 311 18   16.421

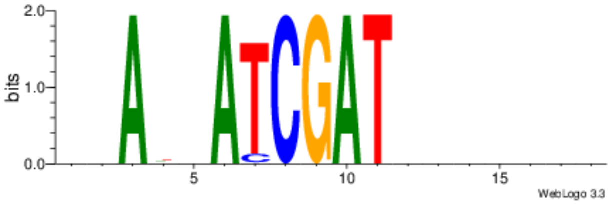
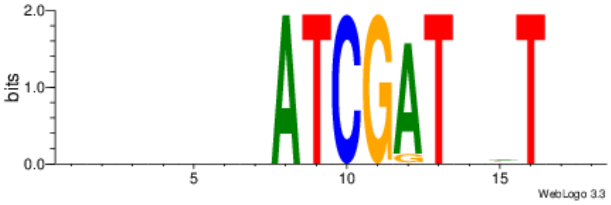

AGT 472 28   16.310

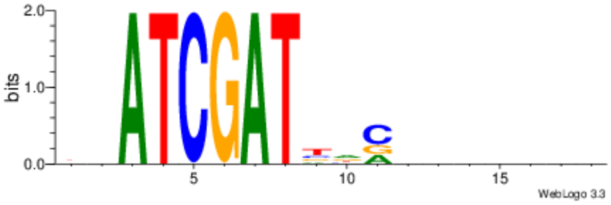
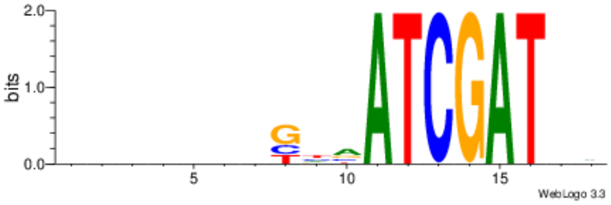

AGT 285 18   15.035

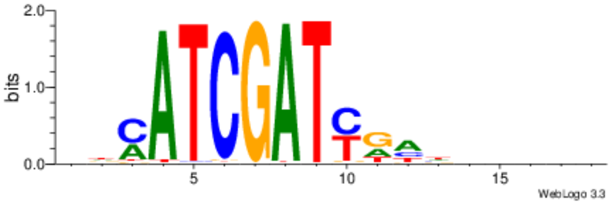
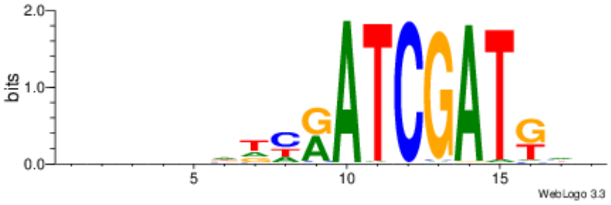

AGT 326 21   14.864

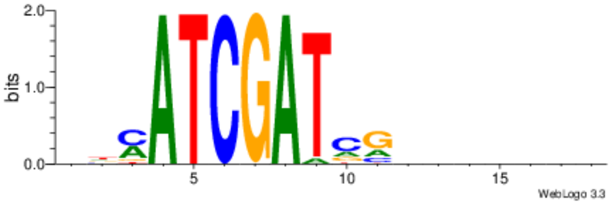
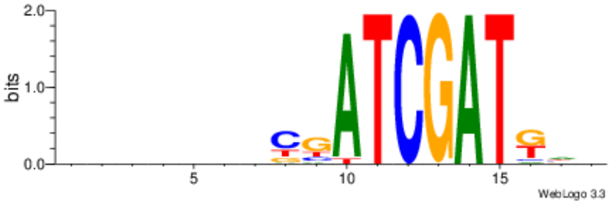

AGT 279 20   13.333

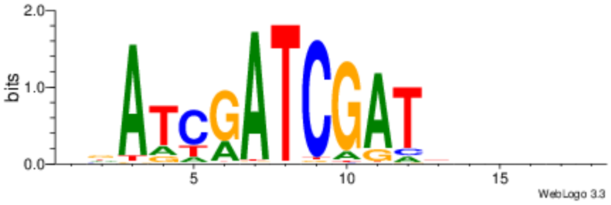
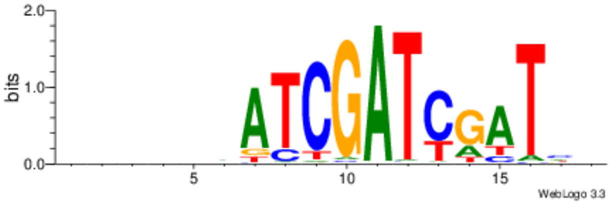

Barcode ATA: 5 nM MBP-Miz1-ZF, 100 mM salt

BC   FG BG Enrichment Forward motif   Reverse comp. motif

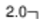

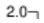

ATA 97 4 19.600

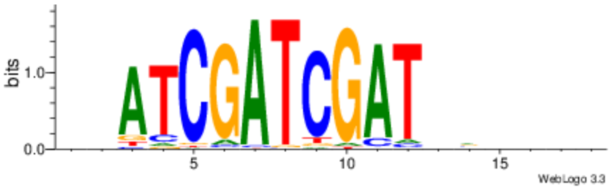

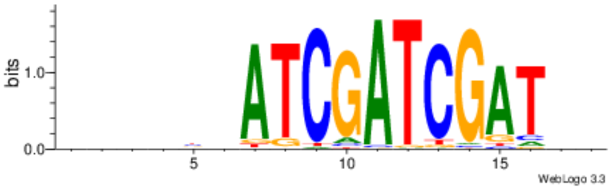

ATA 287 17 16.000

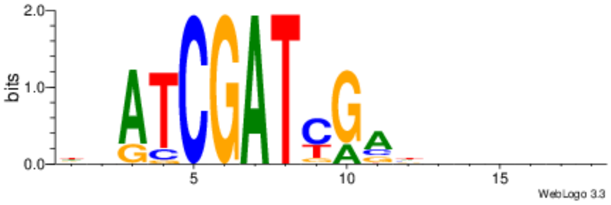

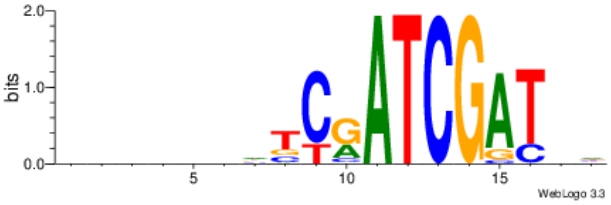

ATA 312 21 14.227

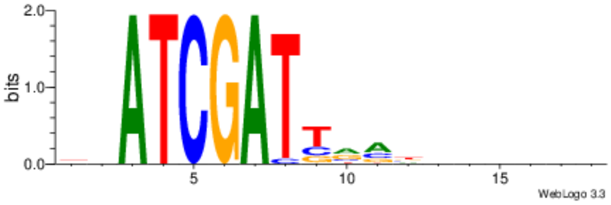

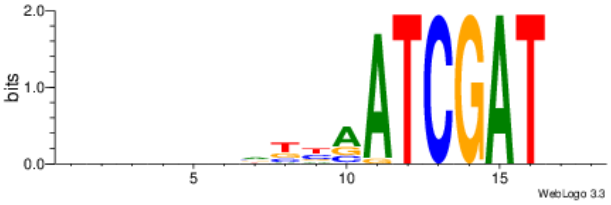

ATA 60 4 12.200

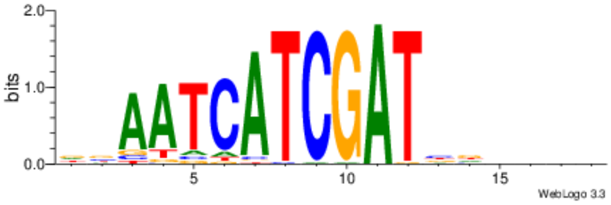

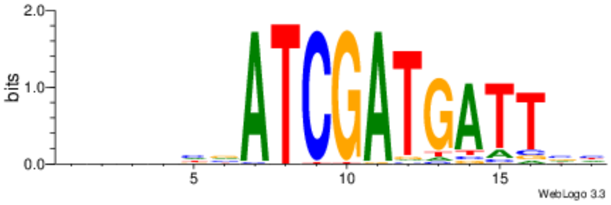

ATA 153 13 11.000

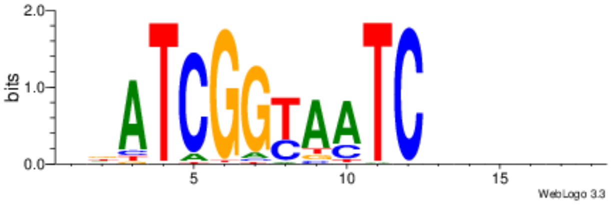

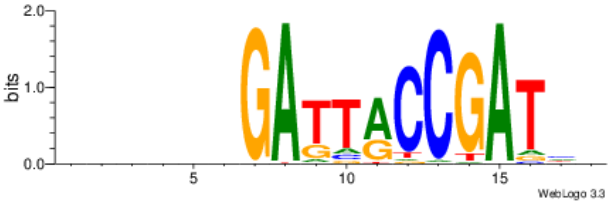

Barcode ATC: 120 nM MBP-Miz1-ZF, 100 mM salt

BC FG BG Enrichment Forward motif — Reverse comp. motif

ATC 128 11 10.750

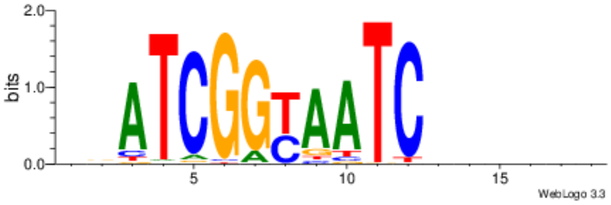

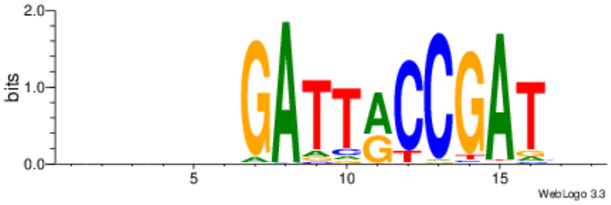

ATC 201 20 9.619

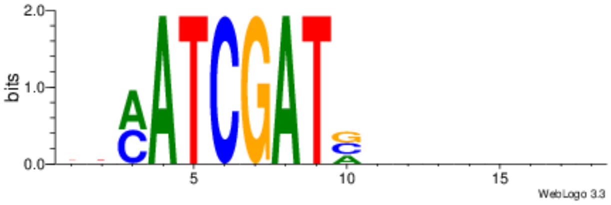

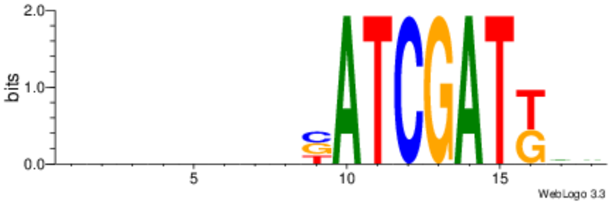

ATC 52 5 8.833

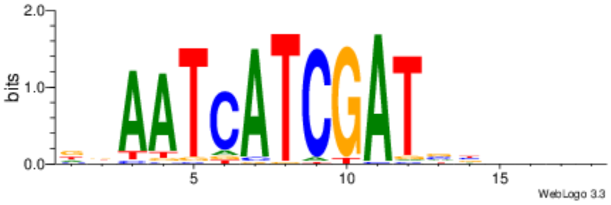

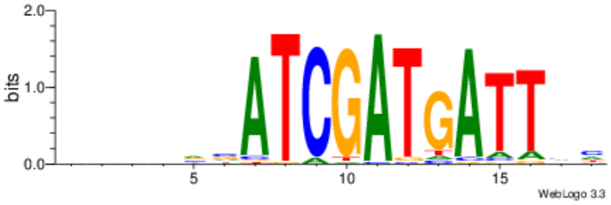

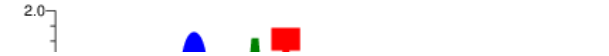

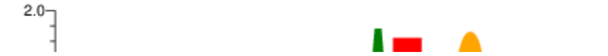

ATC 248 31 7.781

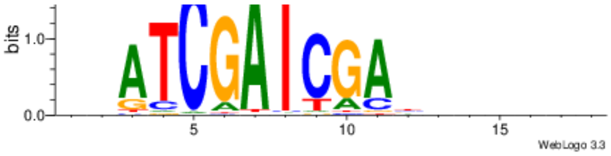

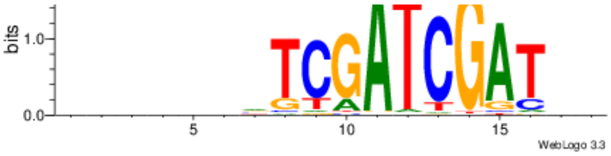

ATC 254 36 6.892

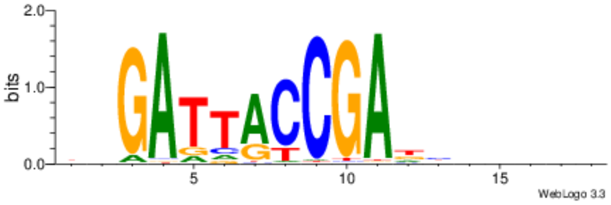

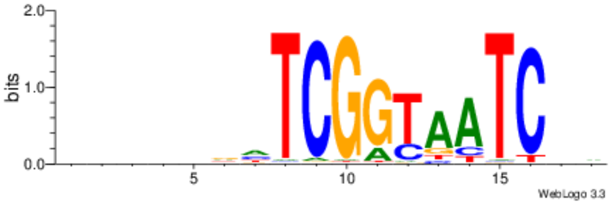

Supplement: Figure S2 — BnS motifs obtained using MBP-Miz-1-ZF protein. Each of the five barcodes refers to a separate BnS experiment using the stated concentrations of protein and salt. For each barcode, the top five highest scoring motifs are reported. (PDF) [file pone.0101151.s002.pdf]
